# miR-486 is modulated by stretch and increases ventricular growth

**DOI:** 10.1172/jci.insight.125507

**Published:** 2019-09-12

**Authors:** Stephan Lange, Indroneal Banerjee, Katrina Carrion, Ricardo Serrano, Louisa Habich, Rebecca Kameny, Luisa Lengenfelder, Nancy Dalton, Rudolph Meili, Emma Börgeson, Kirk Peterson, Marco Ricci, Joy Lincoln, Majid Ghassemian, Jeffery Fineman, Juan C. del Álamo, Vishal Nigam

**Affiliations:** 1 Division of Cardiovascular Medicine, Department of Medicine, UCSD School of Medicine, San Diego, California, USA.; 2 Institute of Medicine, Department of Molecular and Clinical Medicine, the Wallenberg Laboratory and Wallenberg Centre for Molecular and Translational Medicine, University of Gothenburg, Gothenburg, Sweden.; 3 Division of Cardiology, Department of Pediatrics, UCSD School of Medicine, San Diego, California, USA.; 4 Department of Mechanical and Aerospace Engineering, UCSD, San Diego, USA.; 5 Department of Pediatrics, UCSF School of Medicine, San Francisco, USA.; 6 Division of Cardiothoracic Surgery and; 7 Division of Pediatric Surgery, Department of Surgery, Carver College of Medicine, University of Iowa, Iowa City, Iowa, USA.; 8 Center for Cardiovascular Research, Nationwide Children’s Hospital, Columbus, Ohio, USA.; 9 Department of Chemistry and Biochemistry, UCSD, San Diego, USA.; 10 Division of Cardiology, Department of Pediatrics, University of Washington School of Medicine, Seattle, Washington, USA.; 11 Seattle Children’s Research Institute, Seattle, Washington, USA.

**Keywords:** Cardiology, Cell Biology, Cardiovascular disease, Noncoding RNAs, Proteomics

## Abstract

Perturbations in biomechanical stimuli during cardiac development contribute to congenital cardiac defects such as hypoplastic left heart syndrome (HLHS). This study sought to identify stretch-responsive pathways involved in cardiac development. miRNA-Seq identified miR-486 as being increased in cardiomyocytes exposed to cyclic stretch in vitro. The right ventricles (RVs) of patients with HLHS experienced increased stretch and had a trend toward higher miR-486 levels. Sheep RVs dilated from excessive pulmonary blood flow had 60% more miR-486 compared with control RVs. The left ventricles of newborn mice treated with miR-486 mimic were 16.9%–24.6% larger and displayed a 2.48-fold increase in cardiomyocyte proliferation. miR-486 treatment decreased FoxO1 and Smad signaling while increasing the protein levels of Stat1. Stat1 associated with Gata-4 and serum response factor (Srf), 2 key cardiac transcription factors with protein levels that increase in response to miR-486. This is the first report to our knowledge of a stretch-responsive miRNA that increases the growth of the ventricle in vivo.

## Abstract 2

miR-486 is part of a mechano-responsive molecular pathway that increases cardiac growth.

## Graphical abstract

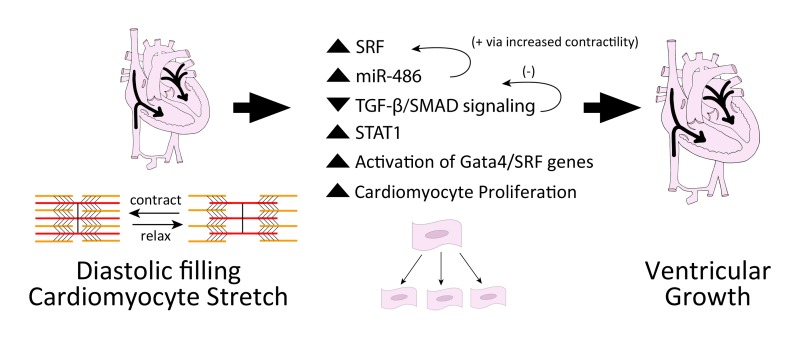

## Introduction

**Figure 1:**
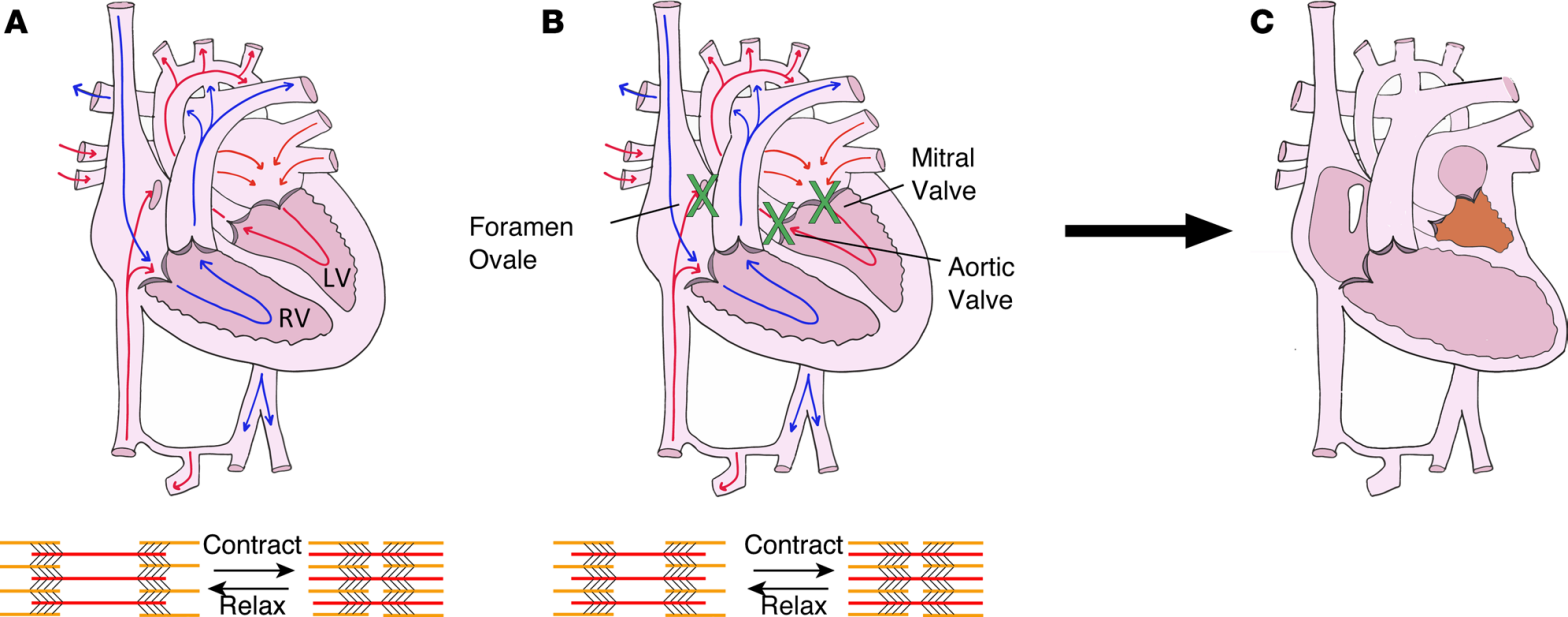
Perturbations in biomechanical stretch in utero can cause HLHS. (**A**) Drawing of a normal fetal heart. During diastolic filling, cardiomyocytes are stretched, and they shorten during systolic contraction. (**B**) In utero stenosis (narrowing) of the foramen ovale, mitral valve, and/or aortic valve, which are represented by the green crosses, results in impaired filling or emptying of the left ventricle (LV). Disruption of LV diastolic filling causes the LV cardiomyocytes to experience decreased stretch as compared with normal hearts. Decreased cyclic stretch impairs the proliferation and growth of cardiomyocytes. As result of the attenuation of stretch-mediated stimulation of ventricular cardiomyocytes, the patients have impaired LV growth. This perturbation in biomechanical loading results in children being born with hypoplastic left heart syndrome (HLHS). (**C**) In HLHS hearts, the patient is born with a diminutive LV that is not large enough to pump enough blood to support the patient’s body.

Biomechanical stretch stimuli are critically important in the heart, where ventricular cardiomyocytes are stretched every heartbeat during diastolic filling. Disruption of biomechanical stretch in utero can result in severe cardiac defects such as hypoplastic left heart syndrome (HLHS). Patients with foramen ovale or mitral/aortic valve stenosis have impaired filling and/or emptying of the left ventricle (LV) in utero and frequently develop hypoplastic LVs that are unable to support the postnatal systemic circulation (Figure 1 and refs. 1–3). Given poor outcomes and challenges associated with the current management of HLHS (4), there is an unmet need to understand the etiology of perturbed growth in utero and to identify small molecules that improve LV growth in HLHS patients. However, while existing animal models (5–11) and computational studies (12) support the theory that HLHS results from perturbed biomechanical stimuli in utero, there is a paucity of data regarding molecular responses to biomechanical stretch.

miRNAs are small noncoding RNAs that bind mRNAs to attenuate mRNA’s translation and/or stability, thereby modulating protein levels of the target genes. To our knowledge, only two publications (13, 14) have reported unbiased miRNA profiling of cardiac cells exposed to stretch. In this report, we demonstrate that miR-486 is a stretch-responsive miRNA that is sufficient to increase ventricular growth, cardiomyocyte proliferation, and Stat1 levels in vivo. miR-486 is enriched in striated muscle (15–17) but little is known about its role in the heart. We have identified miR-486 as being upregulated by stretch in vitro and in vivo, and it tends to be increased in the right ventricles (RVs) of HLHS patients. Specifically, an unbiased miRNA profiling of embryonic mouse cardiomyocytes (EMCMs) exposed to cyclic stretch in vitro demonstrated that miR-486 levels were increased by stretch as compared with static controls. This upregulation by stretch was confirmed by qPCR on samples from dilated sheep RVs as compared control RVs. There was also a trend of increased miR-486 levels in HLHS RVs, which experience increased stretch, as compared with controls. In vitro, cardiomyocytes treated with miR-486 were more contractile than scramble-treated controls and displayed reduced levels of FoxO1 and Smad2/3, known targets for this miRNA (18–20). Increasing miR-486 in newborn mice for 3 days was sufficient to significantly increase ventricular growth and cardiomyocyte proliferation. Moreover, proteomic screening of miR-486–treated murine hearts, as compared with control hearts, identified Stat1 as one of the proteins most upregulated by miR-486 treatment. Given these data, we propose what we believe to be a novel molecular mechanism by which miR-486 promotes stretch-responsive cardiac growth.

## Results

### miR-486 levels are increased in response to stretch in vitro, in patient samples, and in an animal model of ventricular dilation.

To identify stretch-responsive miRNAs in an unbiased manner, we performed miRNA-Seq on RNA isolated from EMCMs exposed to cyclic stretch in vitro (16% at 1 Hz for 24 hours) versus static controls. miRNA-Seq identified 34 miRNAs as being stretch responsive based upon a FDR < 0.05 (Table 1 and Supplemental Table 1; supplemental material available online with this article; https://doi.org/10.1172/jci.insight.125507DS1). Eleven of these miRNAs were upregulated by stretch, while the remaining 23 were downregulated.

**Figure 2:**
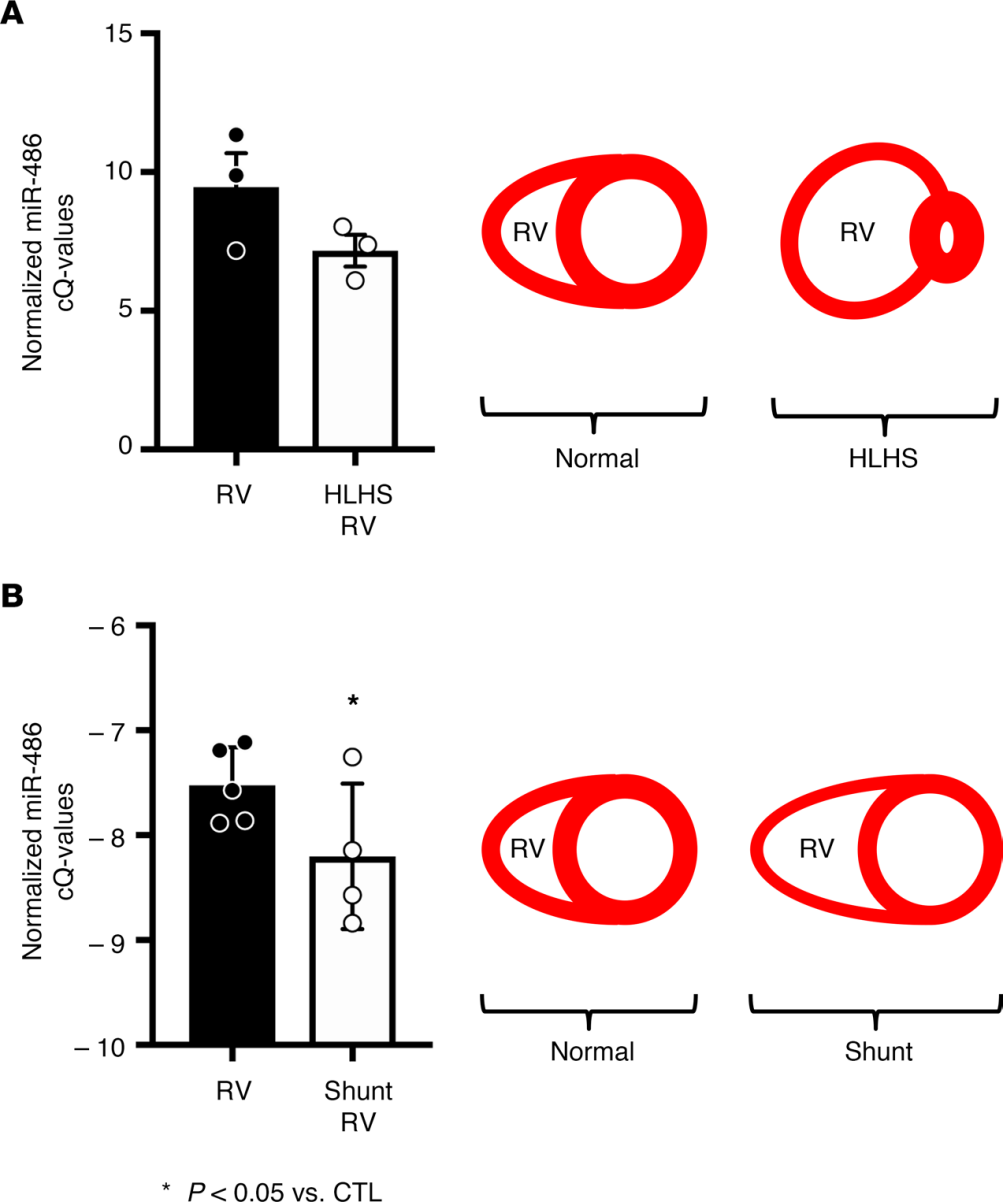
miR-486 levels are increased in HLHS patient RVs and shunted sheep right ventricles. (**A**) In newborn HLHS patients, the right ventricle (RV) cardiomyocytes experience increased stretch, since the RV is facing increased volume and pressure loading. Based upon qPCR array data, miR-486 levels are up 4.9-fold (corresponding to average control cQ = 9.46 vs. average HLHS cQ = 7.17) in HLHS RVs (*P* = 0.08, as determined by 1-tailed *t* test). A full qPCR array data set is included in the Supplemental Data (Supplemental Table 2). (**B**) Sheep with significant pulmonary overcirculation represent a useful in vivo model of increased ventricular stretch. A large unrestrictive aortopulmonary shunt is surgically implanted in late gestation fetal life. After birth, the presence of this shunt continues to expose the RV to systemic-level afterload. As a result of this increased afterload, the RV dilates (66) and ventricular cardiomyocytes experience increased stretch. Shunted sheep RVs have 60% more (corresponding to average control cQ = –7.52 vs. average shunt RV cQ = –8.2) miR-486 as compared with sham-operated RVs (*P* = 0.049 as determined by 1-tailed *t* test). *P < 0.05 vs. control RV.

These data were compared with miRNA profiling, performed by miRNA qPCR array, of RV samples from neonatal HLHS patients undergoing cardiac surgery. In fetal and newborn HLHS patients, the RV experiences increased stretch, since it is providing both the pulmonary and systemic blood flows. RVs of HLHS patients generate the systemic blood pressure, which is significantly higher than pulmonary arterial pressures. Hence, HLHS RVs are increasingly stretched due to the elevated pressure load. Increased blood volume and pressure load caused the RV to become stretched and dilated (Figure 2A). As a result of this physiology, RV samples from neonatal HLHS patients can provide insight into the stretch-responsive molecular changes that occur in human hearts. miRNA qPCR array analysis was performed on RV samples removed from HLHS patients during the initial surgical palliation, which can involve placement of a shunt connecting the RV to the pulmonary artery (PA), as compared with control RV tissue. For several years, albeit not current practice, the surgeon would remove a small piece of the RV in order to connect the RV/PA shunt to the RV; therefore, there were a limited number of HLHS patient samples available for the miRNA analysis. Given the small sample size (*n* = 3 for both groups), we focused on miRNAs that were changed with a *P* value of less than 0.1. miR-486 was the only miRNA that was modulated by stretch in the same direction in the HLHS patient RV compared with control RV qPCR array data set, as the in vitro miRNA-Seq data of stretched versus static cardiomyocytes. Specifically, there was a trend for miR-486 levels to be 4.9-fold higher in the HLHS RVs than in control RVs (*P* = 0.08, average control cQ = 9.46 vs. average HLHS cQ = 7.17; Figure 2A and Supplemental Table 2). Furthermore, we decided to investigate miR-486, because it has been demonstrated to have a role in striated muscle (15–17) and because of its role in attenuating TGF-β signaling (16, 21), which has been shown to repress stretch-mediated cardiomyocyte proliferation and growth (22).

To confirm if stretch was sufficient to increase miR-486 levels in vivo, we compared miR-486 levels in control and dilated sheep RVs. Sheep with an aortopulmonary shunt develop RV dilation secondary to elevated PA pressures (23). This dilation results in increased stretch of the RV cardiomyocytes. Shunted RVs had 60% more miR-486 as compared with sham-operated RVs (*P* < 0.05; average control cQ = –7.52 vs. average shunt RV cQ = –8.2; Figure 2B). Based upon our in vitro, patient, and sheep data — we determined that miR-486 levels are increased by stretch.

### miR-486 is sufficient to increase cardiomyocyte contractility in vitro.

**Figure 3:**
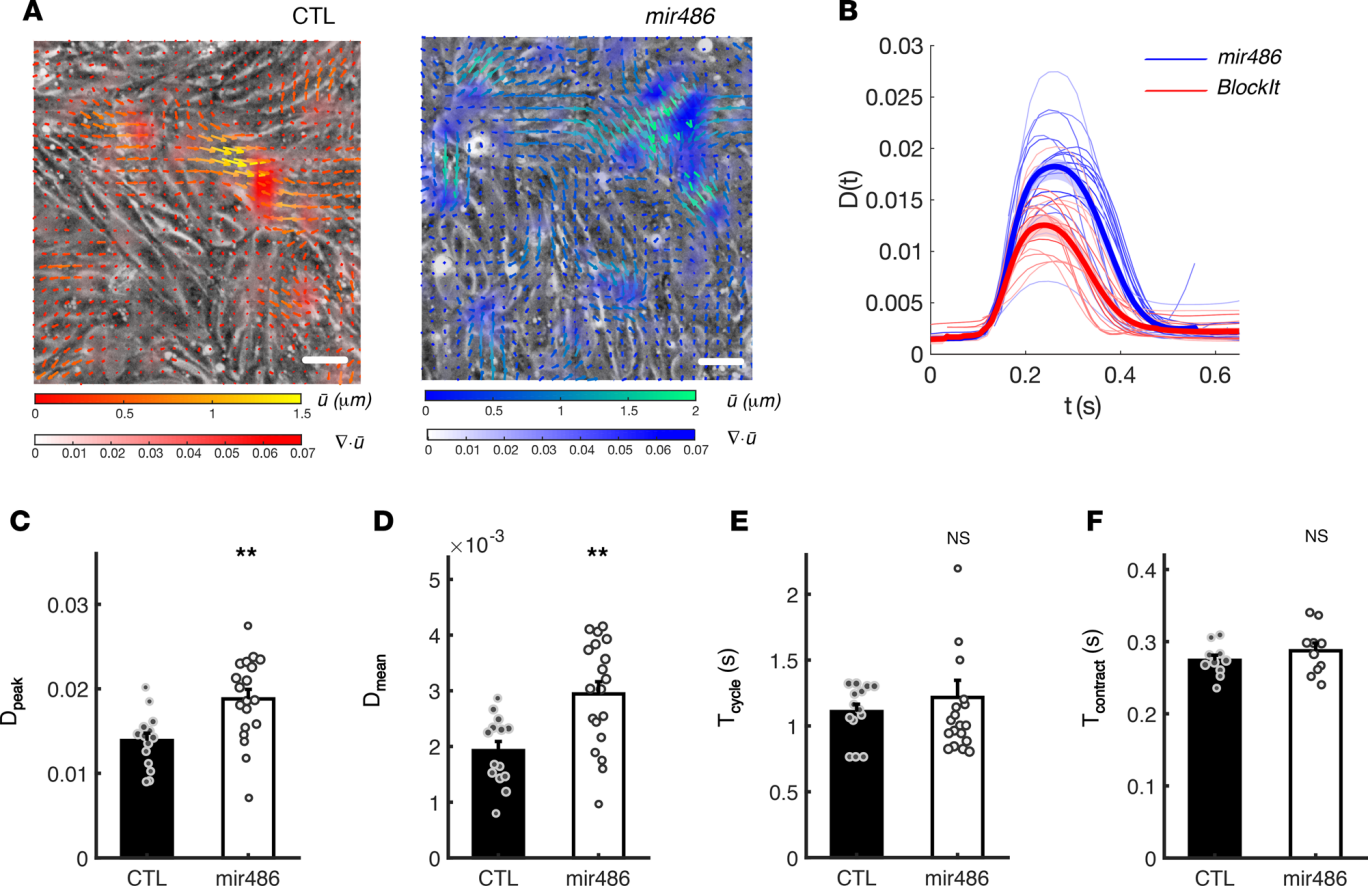
miR-486 increases the contractile function of cardiomyocytes in vitro. (**A**) Instantaneous phase-contrast snapshots of confluent beating EMCMs live stained with wheat germ agglutinin (WGA) Alexa Fluor 488 conjugate. Left: scramble control. Right: miR-486 mimic–treated cardiomyocytes. Vectors represent the cellular displacement field 
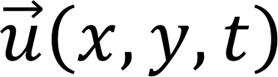
 measured by tracking the motion of WGA speckles. Color mapping represents the absolute value of the divergence of the displacement field, 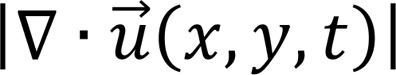, which is a nondimensional quantity that represents the relative change of cell area due to contractility. Its spatial average 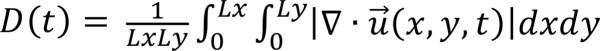 provides a temporal tracing of cellular contractility for each sample. (**B**) Measured tracings of contractility *D(t)* of scramble control (red, *n* = 14) and miR-486 mimic–treated cardiomyocytes (blue, *n* = 19). Thick lines represent the average for each group, while the shaded ribbons span the SEM. (**C**) miR-486–treated cardiomyocytes generate higher peak contractility *D_peak_* as compared with scramble controls (0.014 ± 0.001 s vs. 0.019 ± 0.001 s, *P* = 0.003, as determined by *t* test). (**D**) miR-486–treated cardiomyocytes generate higher time-averaged contractility *D_mean_*, as compared with scramble controls (1.9 × 10^–3^ ± 2 × 10^–4^ vs. 2.9 × 10^–3^ ± 2 × 10^–4^, *P* = 0.002, as determined by *t* test). (**E** and **F**) The scramble control and miR-486 cardiomyocytes have similar beating periods *T_cycle_* (1.11 ± 0.06 s vs. 1.21 ± 0.13 s, *P* = 0.51, as determined by *t* test) and contraction time *T_contract_* (0.27 ± 0.01 s vs. 0.29 ± 0.01 s, *P* = 0.31, as determined by *t* test). **P < 0.01 vs. control.

In order to examine the effects of increased miR-486 levels on cardiomyocyte contractility in vitro, we transfected confluent EMCM cultures with either miR-486 mimic or scramble control (BlockIt) and quantified their beating motion using in-house image analysis methods (Figure 3A and ref. 24). On average, the temporal profiles of contractility derived from these measurements [*D(t),* Figure 3B] experienced similar temporal dynamics, but the miR-486 profiles (*n* = 19) were above control ones (*n* = 14). Statistical analysis of these data showed that miR-486–treated EMCMs generated 35% higher peak contractility (*P* = 0.003; Figure 3C) and 52% higher average contractility (*P* = 0.002; Figure 3D) compared with controls. Beating periods and contraction times for miR-486–treated EMCMs, however, remained unchanged (Figure 3, E and F; *P* = 0.51 and *P* = 0.31, respectively). Overall, these data demonstrate that miR-486 can improve the contractile function of cardiomyocytes.

### miR-486 is sufficient to increase left ventricular growth and cardiomyocyte proliferation in vivo in newborn mice.

**Figure 4:**
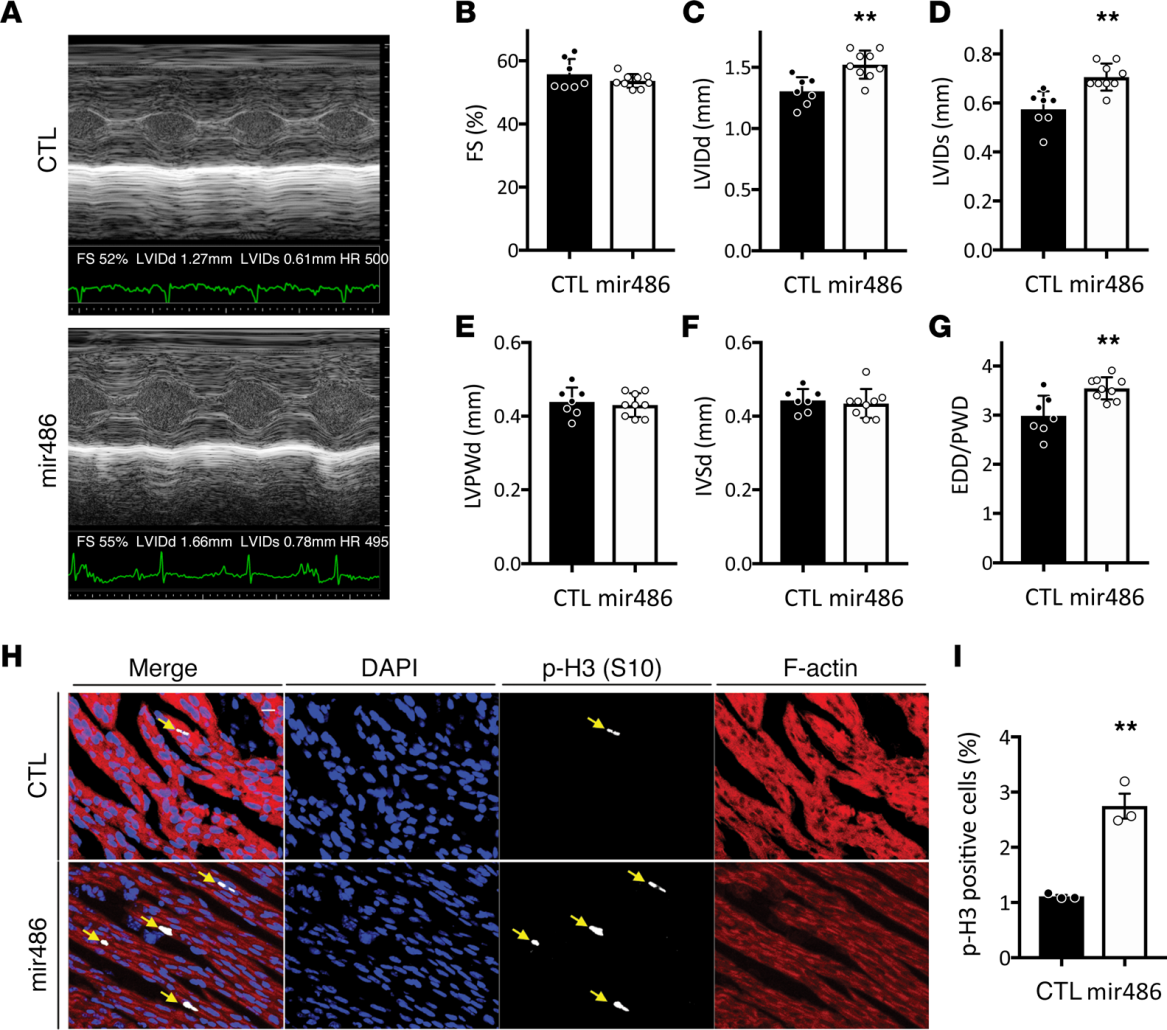
miR-486 is sufficient to increase left ventricular growth and cardiomyocyte proliferation in neonatal mice. Echocardiograms were performed on neonatal mice 3 days after they were treated with miR-486 mimic or scramble control. (**A**) Representative M-mode echocardiography images of scramble BlockIT control– (CTL) or miR-486–treated neonatal mouse hearts used to calculate cardiac parameters (in **B**–**G**). (**B**) Fractional shortening (FS) was not changed between neonatal mice treated with either BlockIT (CTL) or miR-486. (**C** and **D**) LV internal dimension at the end of diastole (LVIDd; **C**) or in systole (LVIDs; **D**) were increased in the miR-486 mice by 16.9% (1.52 mm vs. 1.3 mm, **P < 0.01, as determined by *t* test) and 24.6% (0.71 mm vs. 0.57 mm; **P < 0.01, as determined by *t* test), respectively. (**E** and **F**) LV posterior wall thicknesses (LVPWd; **E**) and interventricular septal thicknesses (IVSd; **F**) during diastole were unchanged between BlockIT (CTL) and miR-486–treated neonatal mice. *n* = 7 control, *n* = 9 miR-486 treated. (**G**) End-diastolic diameter/posterior wall dimension (EDD/PWD) was increased by 20.7% in miR-486 mice (3.61 vs. 2.99, *P* < 0.005, as determined by *t* test). (**H** and **I**) Cardiomyocyte proliferation was quantified from cardiac sections of miR-486– or scramble control–treated (CTL-treated) mice using phospho-Histone H3 (highlighted by arrows; DAPI and F-actin/phalloidin as counterstain; **H**). Magnification of cardiac sections = ×20. Quantification of the ratio of positive phospho-Histone H3 (S10) versus DAPI-stained nuclei (**I**). miR-486 hearts exhibited a 2.48-fold increase in cardiomyocyte proliferation (**P < 0.01, as determined by *t* test, *n* = 3).

To examine effects of increased miR-486 levels on the heart in vivo, we treated newborn mice with a systemically delivered miR-486 mimic or scramble control (CTL, BlockIT). Echocardiographic data (Figure 4) demonstrated that miR-486–treated mice (*n* = 9) had a 16.9% larger end-diastolic LV internal diameter (LVIDd, 1.52 mm vs. 1.30 mm, *P* < 0.01) and a 24.6% larger end-systolic LV internal diameter (LVIDs, 0.71 mm vs. 0.57 mm, *P* < 0.01) than controls (*n* = 7) 3 days after treatment. In addition, end-diastolic diameter/posterior wall dimensions (EDD/PWD) were 20.7% larger in miR-486 mice (3.61 vs. 2.99, *P* < 0.01), and the end-diastolic LV dimension/body weight ratio was increased by 12.5% (0.36 vs. 0.32, *P* < 0.05). Calculated LV mass was increased by 17.7% (10.36 mg vs. 8.80 mg, *P* < 0.05). The calculated LV mass–to–body mass ratio was also increased in miR-486–treated mice by 15.4% (2.47 mg/gm vs. 2.14 mg/gm, *P* = 0.05). There were no significant differences in ventricular wall thicknesses (LVPWd), heart rate, or fractional shortening (%FS) between the two groups (Figure 4 and Supplemental Table 3). The observed increases in LV size without changes in wall thicknesses support the finding that increasing miR-486 is sufficient to increase ventricular growth in vivo.

### miR-486 promotes cardiomyocyte proliferation in vivo.

In order to study the cardiomyocyte proliferation rate in miR-486 hearts, we stained cardiac sections for phalloidin, DAPI, and phospho-histone h3 (S10). Phalloidin staining allowed us to identify cardiomyocytes based the actin-staining pattern. DAPI was used to identify the nuclei. Phospho-histone h3 staining demarcated proliferating cells. Histological examination of the hearts demonstrated that the miR-486–treated mice had a 2.48-fold increase in cardiomyocyte proliferation (2.75% vs. 1.11% *P* = 0.002, *n* = 3; Figure 4, H and I).

### miR-486 increases Stat1 protein levels in vivo and in vitro.

**Figure 5:**
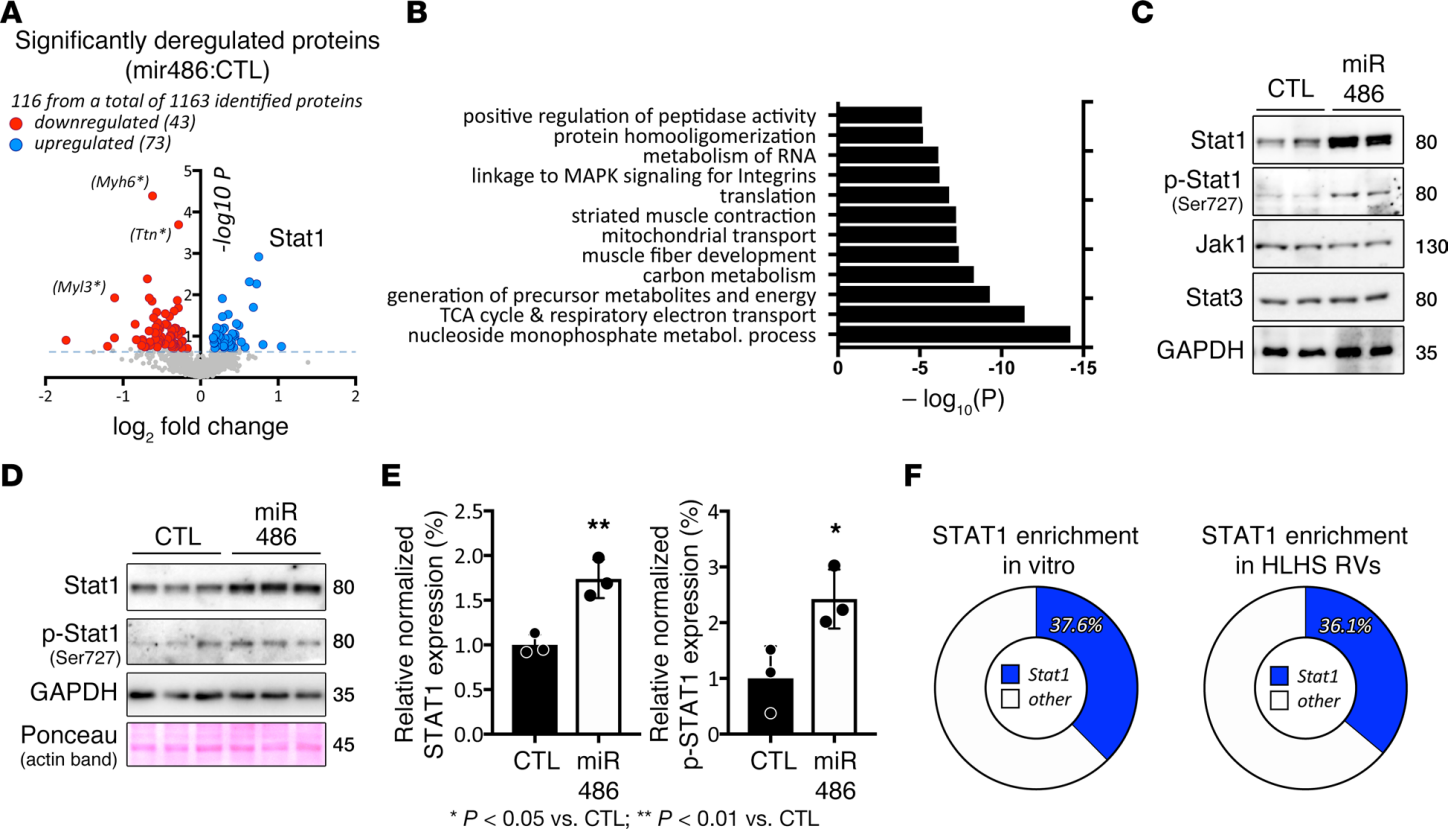
miR-486 is sufficient to increase Stat1 levels in vivo and in vitro. (**A**) Volcano plot showing the results of a mass spectrometry proteome comparison of proteins from miR-486 mimic–treated hearts as compared with scrambled BlockIT controls. Stat1 was one of the most upregulated proteins. Sarcomeric proteins highlighted by asterisks were found dysregulated only in iTRAQ analysis but not in immunoblots (Supplemental Figure 1, C and D) (**B**) Pathway enrichment analysis of significantly changed proteins. (**C**) Immunoblot analysis of heart lysates from miR-486– or BlockIT-treated (CTL) neonatal mice demonstrates that total Stat1 levels are increased in miR-486-treated mice. Of note, miR-486 hearts did not demonstrate alterations in total Jak1 or Stat3 levels. (**D** and **E**) Isolated cardiomyocytes transfected with miR-486 mimic have significantly higher total normalized Stat1 (1.74 ± 0.1249, *n* = 3, *P* = 0.006, as determined by *t* test [*P < 0.05, **P < 0.01 vs. control]) and p-Stat1 Ser727 levels (2.425 ± 0.3063, *n* = 3, *P* = 0.035, as determined by *t* test) as compared with scramble control–treated (BlockIT) cells. Protein levels were normalized to GAPDH. (**F**) Pie chart showing that 37.6% of genes upregulated in cardiomyocytes exposed to cyclic stretch in vitro are predicted have Stat1-binding sites (left), and pie chart showing that 36.1% of genes upregulated in the RVs of HLHS patients as compared with control RVs are predicted to have Stat1-binding sites (right).

We performed iTRAQ-based mass spectrometry proteomics (25) to compare protein samples from miR-486 mimic– and scramble control–treated (BlockIT) mouse hearts. We identified 116 proteins as being modulated (73 upregulated, 43 downregulated) by miR-486 mimic treatment with *P* < 0.2 (Figure 5A and Supplemental Table 4). Gene ontology (GO term) enrichment analysis identified muscle fiber development [–log10(*P*) = –7.39] and striated muscle contraction [–log10(*P*) = –7.22] as among the most significant GO terms modulated by miR-486 treatment (Figure 5B). We focused on Stat1, given it was one of the most upregulated proteins in the iTRAQ data (Figure 5A and Table 2) and literature, indicating stretch stimulation of cardiomyocytes activates Stat1 signaling (26, 27).

We confirmed that miR-486 is sufficient to increase Stat1 protein levels by immunoblotting on total protein extracted from treated mouse hearts (Figure 5C). Since phosphorylation of Stat1 is key for the activity of the proteins and transcription of target genes (28, 29), we performed immunoblot analysis for p-Stat1 (Ser727). Indeed, miR-486–treated hearts displayed increased p-Stat1 (Ser727) compared with scramble-treated (BlockIT) controls. Of note, we examined the protein levels for Stat3 and Jak1, since canonically Stat1 is part of a signaling cascade involving Jak1/Stat3. Interestingly, there was no change in total Stat3 or Jak1 levels (Figure 5C). Finally, to test if increased Stat1 levels originate from cardiomyocytes or other tissue types present in hearts, we isolated and transfected neonatal mouse cardiomyocytes with miR-486 mimic or scrambled control (BlockIT). Similar to our in vivo data, transfected cardiomyocytes also significantly increased total Stat1 (1.74 ± 0.1249, *n* = 3, *P* = 0.006) and p-Stat1 (2.425 ± 0.3063, *n* = 3, *P* = 0.03) levels after 3 days (Figure 5, D and E). To further elucidate Stat1’s potential role in modulating the transcriptional response to biomechanical stretch, we looked for enrichment of predicted Stat1-binding sites within 5 kb of the genes activated by stretch in vitro and in HLHS patient samples. oPOSSUM analysis (30) of the genes upregulated by cyclic stretch in vitro (FDR < 0.05, >1.2-fold increase) (22) showed that 37.6% (384 of 1020 genes) were predicted to have at least one Stat1-binding site (*Z*-score 3.4, Fisher score of 49.7) (Figure 5F). Similarly, 36.1% of the genes upregulated in HLHS RVs (31) were predicted to have at least one STAT1-binding site (*Z*-score for Stat1 was 8.9, with a Fisher score of 14.8; Figure 5F).

### miR-486 represses Tgf-β/Smad signaling.

Prior reports from our group demonstrated that TGF-β signaling is repressed in EMCMs stimulated via cyclic stretch (22) and in HLHS patient RVs that experience increased stretch compared with controls (32). TGF-β and Stat1 pathways can act in a repressive manner on each other (33–35), and part of this negative feedback loop involves TGF-β repression of total Stat1 protein levels (36). Moreover, published data have implied a negative feedback circuit between miR-486 and Tgf-β/Smad (16, 21). We sought to further elucidate this mechanism, and the interplay among Tgf-β/Smad, Stat1, and miR-486.

**Figure 6:**
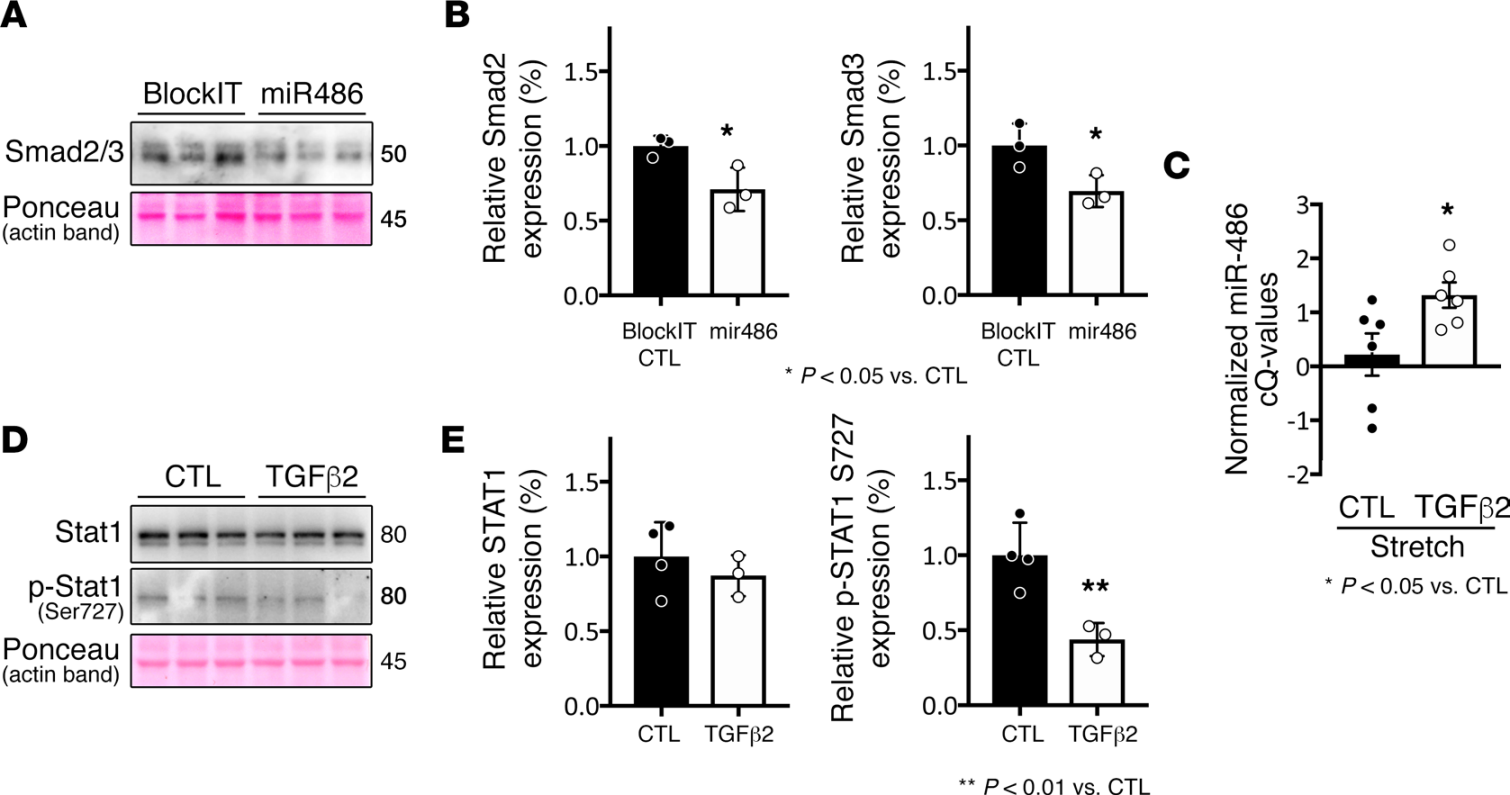
miR-486 modulates Stat1 levels by targeting Tgf-β/Smad signaling. (**A** and **B**) Cardiomyocytes transfected with miR-486 have significantly less Smad2 (0.71 ± 0.08, *n* = 3, *P* = 0.036, as determined by *t* test) and Smad3 levels (0.69 ± 0.06121, *n* = 3, *P* = 0.044, as determined by *t* test), as compared with scrambled controls (BlockIT). Smad levels were normalized to actin. (**C**) The TGF-β2–treated cells have 53.3% less (corresponding to average control cQ = 0.22 vs. average TGF-β2–treated EMCM cQ = 1.32) miR-486, as compared with untreated cells exposed to cyclic stretch (*P* < 0.05, as determined by *t* test). (**D** and **E**) Cardiomyocytes treated with TGF-β2 have significantly decreased p-Stat1 S727 levels (0.44 ± 0.06, *n* = 3, *P* = 0.0099, as determined by *t* test) as compared with untreated controls (BlockIT). Total Stat1 levels were not significantly changed (0.87 ± 0.08, *n* = 3, *P* = 0.4347, as determined by *t* test). Stat1 and p-Stat1 levels were normalized to actin. *P < 0.05, **P < 0.01 vs. control.

First, we examined the feedback between miR-486 and Tgf-β/Smad in cardiomyocytes. Cardiomyocytes treated with miR-486 mimic had significantly lower Smad2 (0.71 ± 0.08, *n* = 3, *P* = 0.03) and Smad3 (0.69 ± 0.06121, *n* = 3, *P* = 0.04) levels as compared with scramble-treated controls (Figure 6, A and B). These data are consistent with recent reports describing direct regulation of Smad2/3 by miR-486 (19, 20). In order to examine if Tgf-β/Smad signaling is involved in the stretch-mediated increase in miR-486 levels, we treated EMCMs with TGF-β2 during cyclic stretch. TGF-β2–treated EMCMs had 53.3% less miR-486 as compared with untreated cells exposed to cyclic stretch (*P* < 0.05, average control cQ = 0.22 vs. average TGF-β2–treated EMCM cQ = 1.32; Figure 6C). These data suggest that the stretch repression of Tgf-β/Smad signaling results in increased miR-486 expression. Cardiomyocytes treated with TGF-β2 have decreased p-Stat1 S727 levels (0.44 ± 0.06, *n* = 3, *P* = 0.0099) as compared with untreated controls (Figure 6, D and E).

### miR-486 increases Gata-4 and Srf levels.

**Figure 7:**
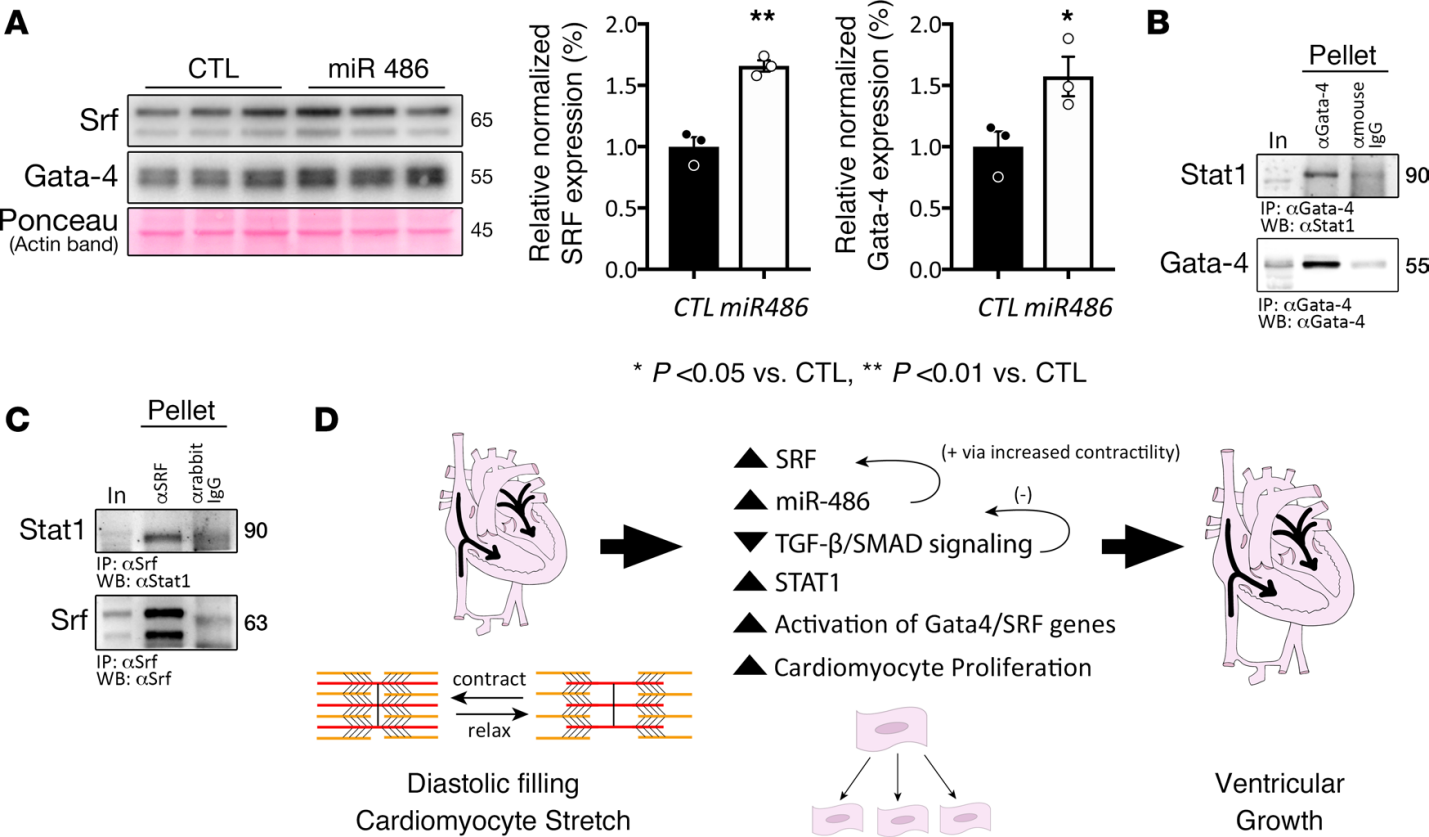
miR-486 modulates cardiac growth through Stat1, Srf and Gata-4. (**A**) miR-486 increases Srf and Gata-4 expression levels in transfected cardiomyocytes after 3 days. Quantification of blots is shown. Srf and Gata-4 levels were normalized to actin. (**B** and **C**) Coimmunoprecipitation experiments using either antibodies directed against Gata-4 (**B**) or SRF (**C**) demonstrate association with Stat1 in cardiac protein extracts. Normal mouse or rabbit IgG was used as negative controls. IN, input; pellet, bound immunocomplexes. (**D**) Outline of proposed mechanism by which stretch increases miR-486 and left ventricular growth. Diastolic filling stretches ventricular cardiomyocytes. This stretch results in increased expression of Srf, which in turn augments expression of miR-486. miR-486 forms a positive feedback loop that increases Srf/Gata-4 and a negative feedback loop with Tgf-β/Smad signaling. Decreased Tgf-β/Smad signaling releases its repression of Stat1. Stat1 coactivates Gata-4/Srf target genes, thereby increasing cardiomyocyte proliferation and ventricular growth. *P < 0.05, **P < 0.01 vs. control.

Besides repressing Tgf-β/Smad signaling, biomechanical stretch also activates cardiac transcription factors Srf (22, 37) and Gata-4 (38), which have key roles in promoting cardiomyocyte proliferation and growth (39–42). Given that stretch increases miR-486 levels, we tested whether miR-486 alone may positively modulate Srf and Gata-4. Indeed, treatment of cardiomyocyte cultures with miR-486 increased Srf (1.658 ± 0.046, *n* = 3, *P* = 0.0019) and Gata-4 (1.573 ± 0.1603, *n* = 3, *P* = 0.047) protein levels compared with BlockIT-treated controls (Figure 7A).

### Stat1 interacts with Gata-4 and Srf in vivo.

Stat1 has been shown to directly interact with Gata-4 and synergistically activate cardiac gene expression with Srf (41, 43, 44). We aimed to validate whether Stat1 associates with Gata-4 in vivo and test for additional association with Srf. Coimmunoprecipitation experiments using endogenous Srf, Gata-4, and Stat1 demonstrate association of the transcription factors in cardiac lysates (Figure 7, B and C), suggesting that Stat1 may form complexes with Srf and/or Gata-4 to modulate cardiac gene expression.

## Discussion

Biomechanical stretch stimuli are important in the development and pathophysiology of the heart, because cardiomyocytes experience stretch during diastolic filling of the cardiac chambers. The role of biomechanical stretch in cardiac development is underscored by congenital cardiac defects, such as HLHS, which result from perturbed biomechanical loading of the ventricle in utero. The driver of this pathology is impaired filling and/or emptying of the LV during cardiac development. Based upon fetal echocardiographic data, patients with narrowed or obstruction of the foramen ovale, mitral valve, and/or aortic valve frequently develop HLHS (1–3). Embryonic sheep, chicken, and zebrafish models with decreased ventricular filling also develop ventricular hypoplasia (5–11). These data, along with finite element modeling of human fetal left ventricular development (12) support the theory that HLHS is the result of perturbed biomechanical stimuli in utero. However, molecular responses to biomechanical stretch are poorly understood. Therefore, it has not been possible to develop noninvasive treatment modalities for this cardiac syndrome, which is associated with substantial morbidity and mortality; 1-year transplant-free survival is 68.7% (4). This study shows for the first time to our knowledge that the stretch-responsive miRNA miR-486 is sufficient to promote ventricular growth, demonstrates that this miRNA is sufficient to increase ventricular growth in vivo, and shows that it is a potentially novel activator of Stat1 expression. These findings provide evidence that miRNAs could be used as a potentially novel treatment to improve the ventricular growth in patients born with ventricular hypoplasia, thereby altering the treatment paradigm for these challenging patients.

Diastolic filling stretches the ventricular cardiomyocytes, thereby increasing expression and signaling of mechanical activated transcription factors Srf (22, 37) and Gata-4 (38). Srf has been shown to control miR-486 transcription (15), and our data suggest that miR-486 in turn may indirectly enhance expression of Srf and Gata-4 through increased contractility and biomechanical stretch in a positive feedback loop (Figure 7D). miR-486 forms a negative feedback loop with Tgf-β/Smad signaling (16, 21), partially by directly acting on Smad2/3 and suppressing their expression (19, 20). This feedback loop may help explain our previous findings that stretch represses Tgf-β/Smad signaling in vitro and in HLHS RVs (22, 32). miR-486–mediated decrease in Tgf-β/Smad signaling releases the TGF-β repression on Stat1 expression (36), which may explain how miR-486 increases Stat1 protein levels. The baseline Tgf-β/Smad activity in the cardiomyocytes may account for why we did not demonstrate signification repression of Stat1 levels in the TGF-β2–treated cells (Figure 6, D and E). Stat1 may promote proliferation via its association with Gata-4 and Srf transcription factors (Figure 7, B and C). Indeed, these results support previous findings that show direct association of Stat1 with Gata-4, which synergistically activates cardiac gene expression together with Srf (43), promoting cardiomyocyte proliferation and growth (39–42). Taken together, we propose a mechanism that outlines how stretch may modulate miR-486 expression to promote cardiomyocyte proliferation and ventricular growth (Figure 7D).

There are some reports in the literature describing other direct targets for miR-486 besides Smad2/3 (19, 20). Specifically, FoxO1 (18, 45) and PTEN (17, 46) were shown to be direct miR-486 targets in various cancer cell lines and tissues other than the heart. However, very little is known whether these targets are also regulated by miR-486 in heart and cardiomyocytes. When investigated in our miR-486–transfected cardiomyocyte cultures, FoxO1 protein levels were significantly downregulated (Supplemental Figure 1, A and B), while PTEN was slightly decreased without reaching statistical significance (*P* = 0.18). These data support the efficacy of our miR-486 transfection in cardiomyocytes and that its effects are comparable to previous reports on direct targets for this miRNA.

Mechanistically, we focused on Stat1 because it was among the most upregulated proteins in the miR-486–treated hearts as compared with scramble control–treated hearts. Although not predicted as a direct target of this miRNA, Stat1 has been previously reported as being stretch responsive (26, 27), similar to miR-486. miR-486’s attenuation of TGF-β may release the TGF-β–mediated repression of Stat1 expression. Furthermore, bioinformatic analyses of the 5 kb flanking the genes upregulated in cardiomyocytes exposed to cyclic stretch in vitro and the RVs of HLHS patients showed significant enrichment of STAT1-binding sites. These data indicate that STAT1, in conjunction with important cardiac transcription factors, such as Gata-4 or Srf, plays a key role in the stretch-mediated increase of important genes involved in cardiac growth.

While there have been some efforts to identify stretch-responsive miRNAs in cardiomyocytes (14, 47, 48), this report identified stretch-responsive miRNAs based upon the correlation of in vitro miRNA-Seq data set with data from patients and an animal model of increased ventricular stretch. We focused on miR-486 because of its roles in striated muscle (15–17) and modulation of TGF-β signaling (16, 21), which represses proliferation, growth, and contraction of cardiomyocytes (22). Additionally, several miRNAs — miR-10a, miR-19a/b, miR-99b, miR-208, miR-335, miR-412, and miR-429 — were significantly modulated by stretch in vitro and were changed in the same direction, with a *P* value of between 0.1 and 0.2 in the HLHS RV qPCR array (Supplemental Table 2). Of note, miR-99b has the potential of being a stretch-responsive miRNA worth studying in more detail, since it is also upregulated in RVs of shunted sheep (49). It is possible that a larger number of patient samples would have allowed us to identify some of these miRNAs as being stretch modulated in vivo. However, the number of patient samples in our study was limited, due to improvements in the surgical palliation of HLHS, by which RV tissue is no longer removed from the patient. As a result, it is not feasible at this time to expand the number of similar neonatal HLHS RV samples.

Echocardiographic data demonstrate that miR-486 is sufficient to increase LV growth in vivo. These data are consistent with growth instead of ventricular dilation, given that the LV internal dimensions at the end of systole and diastole as well as EDD/PW were increased, while the ventricular wall thicknesses were unchanged. Indeed, based upon the increased cardiomyocyte proliferation seen in miR-486–treated mice, it appears that at least a component of the ventricular growth is the result of increased number of cardiomyocytes. While a number of miRNAs have been implicated as modulators of ventricular hypertrophy (50–54), the finding that miR-486 is sufficient to increase the growth of the ventricles in vivo is potentially novel. Furthermore, miR-486–treated mice did not have altered heart rate or systolic function as compared with controls, supporting that miR-486 promotes LV growth and not LV dilation. While our in vitro data supported the in vivo finding that miR-486 treatment does not cause chronotropic alterations, it demonstrated that miR-486 treatment increased cardiomyocyte contractility, even if we could not observe improved LV systolic function in vivo. This partial discrepancy is due to both the miR-486 mimic– and scramble control–treated mice having robust systolic function, so that the LV walls touch during systole (Figure 4A). Therefore, it would not be possible to discern any potential improvement in shortening fraction in response to miR-486 treatment.

Our mass spectrometry proteomics of hearts treated with miR-486 also identified a possible reduction in sarcomeric proteins, titin, myosin (Myh6), and myosin light chain (Myl3) (Figure 5A). However, analysis of sarcomeric protein levels in whole cardiac lysates from miR-486–treated mice did not reveal any significant reduction in the expression of titin, myosin, α-actinin-2, troponin-I, or cardiac actin (Supplemental Figure 1, C and D). The contradictory results between our proteome and immunoblot analyses are likely due to a methodological bias, stemming from the necessary depletion of actomyosin components prior to mass spectrometry that allowed for detection of less abundant nonstructural proteins in our cardiac lysates. This depletion may have more significantly affected titin and its sarcomeric binding partners, including myosin heavy chain 6 (Myh6) (55–57) and its associated myosin light chain (Myl3) (58), in the iTRAQ preparation, as there appears to be a trend toward increased overall titin levels in miR-486 hearts (Supplemental Figure 1C). Moreover, expression of the larger and more compliant N-2BA isoform of titin that would allow for better diastolic filling of the heart seems to be increased. The N-2BA isoform includes additional spring-like elements, such as an enlarged PEVK region, through alternative splicing (59). This ratio shift toward more compliant titin isoforms is similar to what we have previously reported occurring in cardiomyocytes exposed to cyclic stretch in vitro (22). Based upon the immunoblot data, we do not believe that miR-486 decreases titin levels and levels of other associated sarcomeric proteins.

Our data suggest that modulation of levels of miRNA, such as miR-486, in vivo may be a potentially innovative approach to increase ventricular growth in patients with ventricular hypoplasia as an alternative to the current surgical palliation for single ventricle patients. Since HLHS patients have decreased expression of proliferation-related genes (60), and animal models for HLHS were also shown to have decreased cardiomyocyte proliferation (7, 8), efforts to improve cardiomyocyte proliferation may improve the size of the LVs in HLHS patients thereby improving their clinical course. Given the pressing need to develop new treatment modalities, part of our future efforts will be to determine if increased miR-486 is sufficient to increase ventricular growth in animal models of ventricular hypoplasia.

## Methods

### Mouse cardiomyocyte cultures.

Cardiomyocytes from hearts of wild-type embryonic (E16.5) or newborn mice (CD1 background, Charles River) were isolated and cultured as previously described (61, 62).

### Biomechanical stretching of cardiomyocytes.

EMCMs were grown on collagen-I–coated Bioflex plates (BF-3001C, Flexcell International). EMCMs were concurrently exposed to cyclic stretch of 16% at 1 Hz for 24 hours using a Flexcell FX-5000 Tension system (Flexcell International) and to static condition (control) on Bioflex plates (22).

### RNA extraction.

RNA was extracted from the EMCMs using the RNeasy Mini kit (Qiagen). RNA concentration was determined at 260 nm using ND‑1000 (Nanodrop), and RNA integrity was assessed using an Agilent 2100 Bioanalyzer.

### miRNA-Seq.

1 μg of total RNA from 6 stretched and 6 static EMCM samples was used for small RNA library preparation using the TruSeq Small RNA protocol (Illumina), similar to methods that we have previously used (13). All RNA was validated using an Agilent Bioanalyzer, and only samples with an RNA integrity number of >8 were used. Illumina adapters were ligated to each end of the RNA molecule, and a reverse transcriptase reaction was used to create single-stranded cDNA. The cDNA was subsequently PCR amplified using a universal primer and a primer containing 1 of 48 Illumina index sequences (Illumina TruSeq SmallRNA kit).

The small RNA libraries were loaded onto the Illumina cBot Cluster Station, where they bound to complementary adapter oligos grafted onto a proprietary flow cell substrate. Isothermal amplification of the cDNA construct was carried out creating clonal template clusters of approximately 1,000 copies each. The Illumina HiSeq2500 directly sequences the resulting high-density array of template clusters on the flow cell using sequencing by synthesis. Four proprietary, fluorescently labeled, reversible terminator nucleotides were used to sequence the millions of clusters base by base in parallel.

For deep-sequencing reads produced by the Illumina HiSeq2500, low-quality reads were filtered out to exclude those most likely to represent sequencing errors, and adaptor sequences were subsequently trimmed into clean full-length reads formatted into a nonredundant Fasta format. The occurrences of each unique sequence read were counted as sequence tags (the number of reads for each tag reflects relative expression level), and only small RNA sequences of 18–30 nt were retained for further analysis. The miRNA-Seq data has been archived at GEO (GSE120676).

All unique sequence tags that passed above filters were mapped onto the reference mouse genome using the Bowtie2 program (63). The hits were counted using in-house scripts written in Perl and then translating the RNA IDs to gene IDs using the NCBI database. All counts that came from the same gene under that gene ID were added. The edgeR algorithm implemented under the Bioconductor suite was used to obtain expression levels and significance (*P* values, *Q* values) (64).

### Human miRNA profiling.

The HLHS patient and control ventricular samples were obtained under a protocol that has been previously described in detail (32). HLHS RV samples were obtained from neonatal patients at the time of the RV to PA shunt placement. The control RV tissue was obtained from infants (oldest was 135 days old) who died from noncardiac causes. We used the TaqMan Array Human MicroRNA from Applied Biosystems (A v2, V v3). cDNA was generated from 500 ng Trizol-extracted tRNA using the Megaplex RT Primers and TaqMan MicroRNA Reverse Transcription Kit from Applied Biosystems.

50 ng of miRNA reverse transcription product was used for each miRNA qPCR reaction. Samples were normalized to a U6 endogenous control and then used to calculate the 2^–ΔΔCT^ values to determine the relative fold changes.

### Sheep RV.

Late gestation fetal sheep had an aortopulmonary vascular graft placed as previously described (65). Four to six weeks after spontaneous delivery, these lambs have biventricular hypertrophy compared with twin controls (66).

### miRNA qPCR.

RNA was isolated using the RNAeasy kit (Qiagen). qPCR was performed using TaqMan primers (miR-486 primer [ThermoFisher assay 001278], U6 snRNA primer [ThermoFisher assay 001973], miR-103 primer [ThermoFisher assay 000439]). For the sheep samples, miR-103 was used for normalization. For the EMCMs, we used U6 as the endogenous control.

### TGF-β2 treatment.

EMCMs or neonatal mouse cardiomyocytes were treated with 1 ng/ml TGF-β2 (R&D Systems) and then were exposed to stretch conditions for 24 hours, or, to study long-term effects, were cultured in static conditions for 3 days, with daily changes of medium supplemented with 1 ng/ml TGF-β2 before analysis. Untreated cardiomyocytes were used as controls.

### miRNA transfection.

Neonatal mouse cardiomyocytes were transfected with either miR-486-5p mimic (Ambion, MC10546, Invitrogen) or scramble control (BlockIT, 14750-100, Life Technologies) using Escort III (MilliporeSigma) (67) according to the manufacturer’s instructions. Cells were harvested for protein analysis or processed for immunofluorescence staining 72 hours after transfection.

### Contractility assays using dynamic monolayer force microscopy.

EMCMs were transfected with either miR-486 mimic (Invitrogen) or scramble control using Lipofectamine 2000 (67). Contractility was assessed 48 hours after transfection as previously described (24). Phase-contrast image acquisition was performed at 20 Hz using a spinning-disk confocal microscope with ×20 magnification. Time-dependent cell deformation vector maps with respect to diastolic relaxation, 
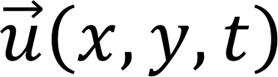
 were derived by in-house particle image velocimetry scripts written for MATLAB. Suitable reference frames representing EMCM diastolic relaxation were selected by performing PIV on consecutive frames to identify the frames with minimal cell velocity. The divergence of 
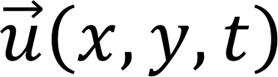
 is a nondimensional variable that quantifies relative changes in area, i.e., contractility, of the EMCMs in the microscopic field of view every time a contraction occurs. Likewise, its square root, i.e., 
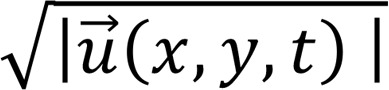
, could be interpreted as a measure of cardiomyocyte shortening during contraction. The spatial average of the divergence, 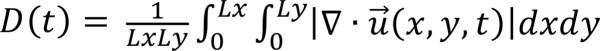, was calculated to obtain temporal tracings of cellular contractility for each experiment. These contractility-versus-time signals were analyzed to calculate chronotropic and inotropic parameters for quantitative and statistical comparisons of samples, such as peak cycle period, *T_cycle_*, the duration of cell contraction within each beating cycle, 
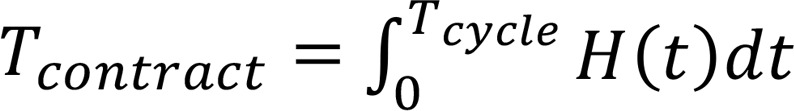
, where *H(t) = 1* if *D(t)* > *0.1D_peak_* and 0 otherwise, peak contractility *D_peak_ = max(D(t))*, and mean contractility 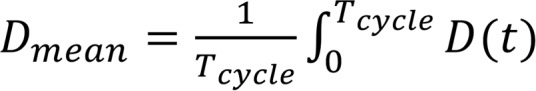.

### In vivo injections.

Newborn mice were injected intraperitoneally with either 10 μg miR-486-5p miRNA mimic (Ambion, MC10546, Life Technologies) or scramble control (BlockIT, 14750-100, Life Technologies) in combination with in vivo Jet PEI (Polyplus transfection, 201-10G), according to the manufacturer’s instructions. Three days following the injection, cardiac functions and morphological parameters of control-, BlockIT-, and miR-486–injected neonatal mice were recorded, before isolation and processing of tissues for further analysis.

### Echocardiography.

For analysis of cardiac functions, postnatal mice were measured by trans-thoracic echocardiography using a FUJIFILM VisualSonics SonoSite Vevo 2100 ultrasound system with a 32- to 55-MHz linear transducer. Fractional shortening was used as an indicator of systolic cardiac function. In addition, heart rate, interventricular septal thickness during diastole, LVIDd, LVIDs, and LVPWd during diastole were measured and analyzed as described previously (68, 69).

### Immunofluorescence staining and analyses.

Acetone-fixed cardiac cryosections were processed for immunofluorescence staining as described earlier (70). Briefly, sections were permeabilized with 0.2% Triton X-100 in 1× PBS for 5 minutes at room temperature, followed by blocking of unspecific binding sites by 5% donkey serum and 1% BSA dissolved in gold buffer (155 mM NaCl, 2 mM EGTA, 2 mM MgCl2, 20 mM Tris-HCl, pH 7.5) for a minimum of 1 hour. After blocking, tissue sections were incubated with primary antibodies diluted in gold buffer overnight at 4°C. Following incubation, specimens were washed 3 times with 1× PBS at room temperature, before incubation of with appropriate secondary antibodies diluted in gold buffer for 1 hour at room temperature. Subsequently, sections were washed 3 times with 1× PBS and mounted using fluorescent mounting medium (DAKO). Immunofluorescently labeled specimens were imaged using a Fluoview 1000 confocal microscope (Olympus) in sequential scanning mode using ×10 air or ×40 oil objectives and zoom rates between ×1 and ×3. Image analysis was performed using ImageJ (NIH).

### Antibodies.

The following primary antibodies were used for immunofluorescence and immunoblot analyses as well as coimmunoprecipitation experiments: Stat1 (Cell Signaling, 9172), p-Stat1 S727 (Cell Signaling, 9177), p-Stat1 Y701 (Cell Signaling, 9167), Smad2/3 (Cell Signaling, 5678); Smad3 and p-Smad3 (Cell Signaling [ref. 22]), SRF (Cell Signaling, 5147), Gata-4 (Cell Signaling, 36966 or Developmental Studies Hybridoma Bank [DSHB]; CRP-GATA-4-1A7, deposited to the DSHB by Protein Capture Reagents Program, produced by JHU/CDI), p-Histone H3 S10 (Cell Signaling, 3377), JAK1 (Cell Signaling, 3332), STAT3 (Cell Signaling, 9404), GAPDH (Santa Cruz Biotechnology, sc-32233), titin T11 (MilliporeSigma, T9030), sarcomeric myosin (DSHB; A4.1025), troponin-I (Cell Signaling, 4002), sarcomeric α-actinin-2 (clone EA-53, Mob 227-05, Diagnostic Biosystems), cardiac actin (Progen Biotechnik, Ac1–20.4.2), PTEN (Cell Signaling, 9188), FoxO1 (Cell Signaling, 2880), normal rabbit IgG (Santa Cruz Biotechnology, sc-2027), normal mouse IgG (Santa Cruz Biotechnology, sc-2025). Secondary fluorescence dye–linked or horseradish peroxidase–linked antibodies were obtained from Jackson Immunoresearch, DAKO, Cell Signaling or Santa Cruz Biotechnology. Fluorescently labeled phalloidin and DAPI was purchased from Molecular Probes (Life Technologies).

### Proteomics.

Proteins were isolated form whole hearts and lysed into ice-cold isolation buffer (300 mM KCl, 30 mM PIPES pH 6.6, 0.5% NP-40, 1× protease inhibitor [Roche], 1× phos-stop [Roche]). Insoluble proteins were removed by centrifugation (18,000 *g*, 10 minutes at 4°C), and the supernatant was diluted 1:4 with ice cold dilution buffer (1× Phos-stop [Roche], 0.5% NP-40, 1 mM DTT). Precipitation of actomyosin components was done by centrifugation (18,000 *g*, 15 minutes at 4°C), and the remaining supernatant was snap-frozen for further analysis by mass spectrometry and immunoblot analyses.

Analysis and identification of peptides via mass spectrometry was done as described previously (71). Immediately prior to mass spectrometry, protein solutions were diluted in TNE buffer (50 mM Tris-HCl pH 8.0, 100 mM NaCl, 1 mM EDTA). RapiGest SF reagent (Waters Corp.) was added to the mix to a final concentration of 0.1% and samples were boiled for 5 minutes. TCEP [Tris (2-carboxyethyl) phosphine] was added to a final concentration of 1 mM, and the samples were incubated at 37°C for 30 minutes. Subsequently, the samples were carboxymethylated with 0.5 mg/ml of iodoacetamide for 30 minutes at 37°C followed by neutralization with 2 mM TCEP (final concentration). Protein samples prepared as above were digested with trypsin (trypsin/protein ratio of 1:50) overnight at 37°C. RapiGest was degraded and removed by treating the samples with 250 mM HCl at 37°C for 1 hour followed by centrifugation at 18,000 *g* for 30 minutes at 4°C. The soluble fraction was then added to a new tube and the peptides were extracted and desalted using C18 desalting tips (Thermo Scientific).

The trypsinized samples (8 samples) were labeled with isobaric tags (iTRAQ8, ABSCIEX) (72), where each sample was labeled with a specific tag to its peptides, as described in the manufacturer’s instructions. Each set of experiments was then pooled and fractionated using high pH reverse-phase chromatography (HPRP-Xterra C18 reverse phase, 4.6 mm × 10 mm, 5-μm particle, Waters). The chromatography conditions were as follows: the column was heated to 37°C, and a linear gradient from 5%–35% B (Buffer A, 20 mM ammonium formate pH 10 aqueous, Buffer B, 20 mM ammonium formate pH 10 in 80% acetonitrile (ACN) water) was applied for 80 minutes at 0.5 ml/minutes flow rate. A total of 42 fractions of 0.5 ml volume were collected. For LC-MS/MS analysis some fractions were pooled to create a final 16 pooled samples. Each of the pooled fractions were analyzed by high-pressure liquid chromatography coupled with tandem mass spectroscopy (LC-MS/MS) using nanospray ionization.

The nanospray ionization experiments were performed using a TripleTof 5600 hybrid mass spectrometer (ABSCIEX) interfaced with nanoscale reversed-phase UPLC (Waters Corp. nano ACQUITY) using a 20-cm 75-μm ID glass capillary packed with 2.5 μm C18 (130 Å) CSHTM beads (Waters Corp.). Peptides were eluted from the C18 column into the mass spectrometer using a linear gradient (5%–80%) of ACN at a flow rate of 250 μl/min for 1 hour. The buffers used to create the ACN gradient were as follows: Buffer A (98% H2O, 2% ACN, 0.1% formic acid, and 0.005% TFA) and Buffer B (100% ACN, 0.1% formic acid, and 0.005% TFA). MS/MS data were acquired in a data-dependent manner in which the MS1 data were acquired for 250 ms at *m/z* of 400–1250 Da, and the MS/MS data were acquired from *m/z* of 50–2,000 Da. The independent data acquisition parameters were as follows: MS1-TOF acquisition time of 250 ms, followed by 50 MS2 events of 48-ms acquisition time for each event. The threshold to trigger a MS2 event was set to 150 counts when the ion had the charge state +2, +3, and +4. The ion exclusion time was set to 4 seconds. The collision energy was set to iTRAQ experiment setting. Finally, the collected data were analyzed using Protein Pilot 5.0 (ABSCIEX) for peptide identifications and peaks (73). Observed protein changes with a *P* value of less than 0.2 were considered significant (74). Bioinformatic enrichment and pathway analysis was done using Metascape (http://metascape.org) (75), Morpheus (https://software.broadinstitute.org/morpheus), and the BioGRID (https://thebiogrid.org) (76).

### Coimmunoprecipitation and immunoblot analysis.

For coimmunoprecipitation experiments, hearts of adult wild-type mice were lysed into ice-cold lysis buffer (150 mM NaCl, 10 mM Tris-HCl pH8, 0.2% NP-40, 0.05% SDS, 1× protease inhibitor cocktail [Roche], 1× phos-stop [Roche], 1 mM DTT). Samples were briefly sonicated at 4°C before centrifugation at 16,000 *g* for 10 minutes at 4°C. Soluble proteins in the supernatant were transferred to fresh tubes, and 1 μg of antibody or normal rabbit or mouse IgG was added to the sample. Following overnight incubation of cardiac lysates with antibodies at 4°C, protein G–coupled magnetic beads (Dynabeads, Life Technologies) were added and samples were incubated on a shaker for 3 hours at 4°C. Beads were washed 3 times with ice-cold 1× PBS supplemented with 0.2% NP-40, and bound immunocomplexes and associated proteins were analyzed by immunoblot analyses. See complete unedited blots in the supplemental material.

Immunoblots of SDS-acrylamide gels or SDS-agarose gels were performed as previously described (77, 78). Briefly, protein samples lysed into sample buffer were boiled for 2 minutes and loaded onto 4%–20% polyacrylamide gradient gels (Bio-Rad) or SDS-agarose gels. Protein samples were run at approximately 120 V for 1 hour and transferred onto nitrocellulose membranes using the wet transfer method. Protein loading levels and successful transfer onto membranes was checked via Ponceau stain and blocked in either 5% milk/TBST or 5% BSA/TBST. Membranes were then probed using primary antibodies diluted in blocking solution. Following overnight incubation at 4°C, membranes were washed 3 times for 5 minutes each with TBST solution, incubated with appropriate secondary antibodies, and diluted into blocking solution for 1 hour at room temperature. After washing of membranes 5 times for 10 minutes in TBST, membranes were developed using SuperSignal West Pico Plus chemiluminescent reagent. Densitometry was performed as previously described (79). If not stated otherwise, reported sample sizes (*N*) represent biologic replicates.

### Prediction of enrichment of transcription factors bindings in stretch-responsive genes.

oPOSSUM analyses (30) were performed to identify enriched transcription factors within the 5 kb flanking the genes identified by stretch in vitro and in the RVs of HLHS patients. We examined the genes upregulated in EMCMs exposed to cyclic stretch in vitro as compared with static control, showing an FDR < 0.05 and more than 1.2-fold increase in mRNA-Seq data (22). In order to identify transcription-binding site enrichment in HLHS patient RVs as compared with control RVs, oPOSSUM analysis was performed on the upregulated genes identified by microarray analysis, with FDR < 0.05 (31).

### Statistics.

For the miRNA-Seq, the edgeR algorithm implemented under the Bioconductor suite was used to obtain significance (*P* values, *Q* values) (64). Observed protein changes with a *P* value of less than 0.2 were considered significant (74). Unless otherwise specified, significance was determined in Excel (Microsoft) or Prism (GraphPad Software, version 7) by performing unpaired 2-tailed *t* test analysis, with a *P* value of less than 0.05 considered as significant.

### Study approval.

The human tissue was collected under an IRB-approved protocol by the University of Miami, Coral Gables, Florida, USA. The mouse experiments were performed under an IACUC-approved protocol at UCSD. The sheep experiments were performed under an IACUC-approved protocol at UCSF.

## Author contributions

SL, IB, and VN designed and performed the experiments and prepared the manuscript. LL, KC, and LH performed some of the experiments. RS, RM, and JCDA performed and analyzed the dynamic monolayer force microscopy experiments. MR collected the HLHS and control patient RV tissue. JL performed and analyzed the miRNA qPCR array data on the human RV tissue. ND performed the echocardiograms. EB helped to analyze data and revise the manuscript. KP helped analyze the echocardiographic data. RK and JF performed the sheep shunt and control surgeries and provided the sheep RV tissue. MG performed the iTRAQ experiment.

## Supplementary Material

Supplemental data

Supplemental Table 1

Supplemental Table 2

Supplemental Table 3

Supplemental Table 4

## Figures and Tables

**Table 2:**
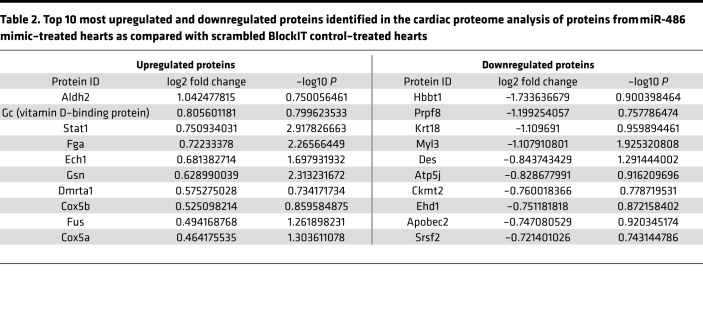
Top 10 most upregulated and downregulated proteins identified in the cardiac proteome analysis of proteins from miR-486 mimic–treated hearts as compared with scrambled BlockIT control–treated hearts

**Table 1:**
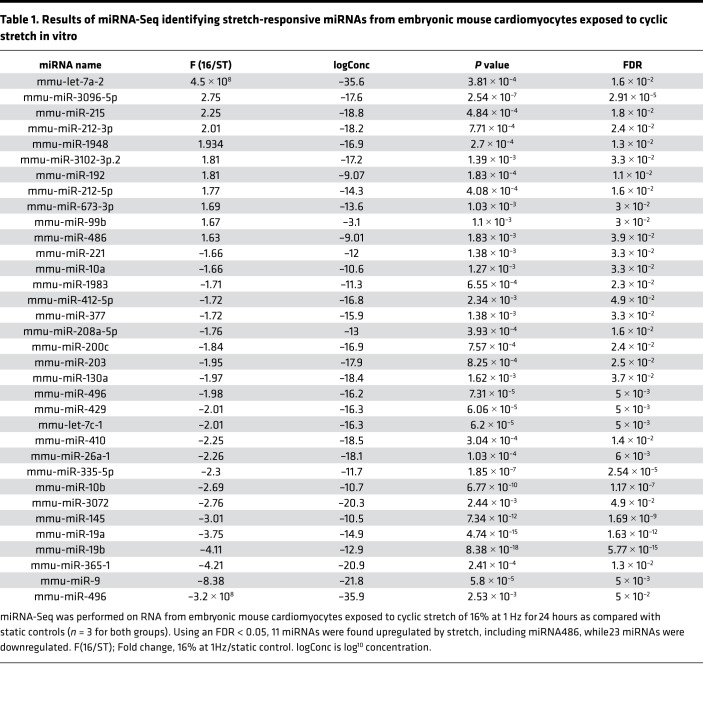
Results of miRNA-Seq identifying stretch-responsive miRNAs from embryonic mouse cardiomyocytes exposed to cyclic stretch in vitro

## Version 1 09/12/2019

In-Press Preview

## Version 2 10/03/2019

Electronic publication
