# Supplementary material for: miR-486 is modulated by stretch and increases ventricular growth
Source: JCI Insight. 2019 Sep 12;4(19):e125507. doi: 10.1172/jci.insight.125507 (PMC6795397; doi:10.1172/jci.insight.125507)

fully unedited gel for Figure 5C
STAT1
pSTAT1-S727
GAPDH

corresponding Ponceau
stained membranes

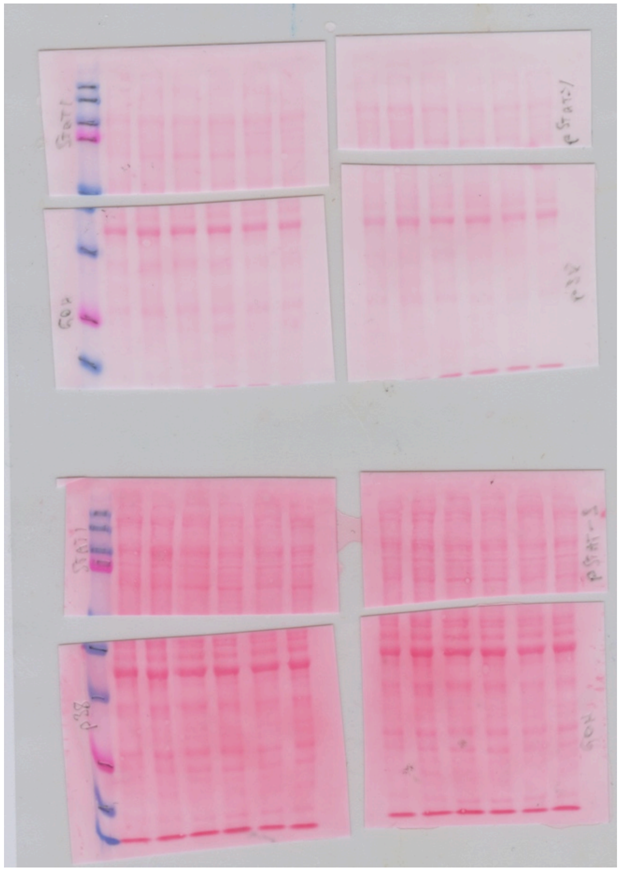

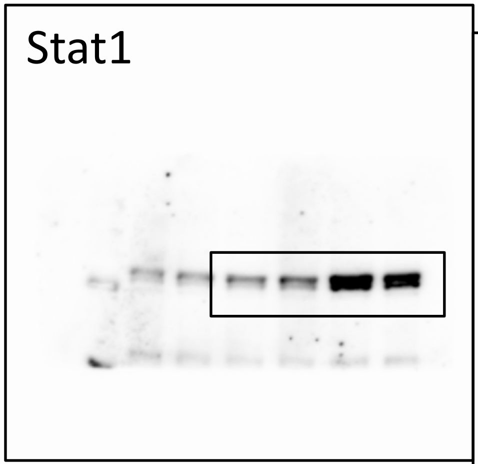

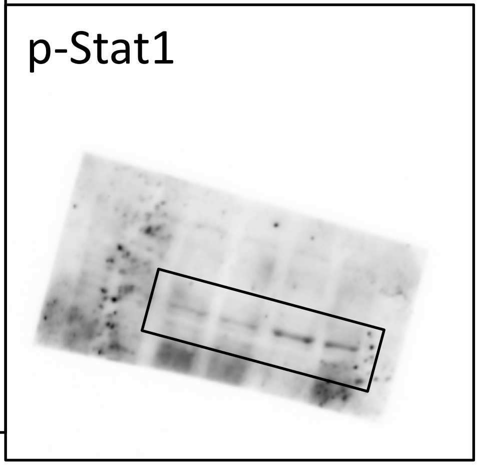

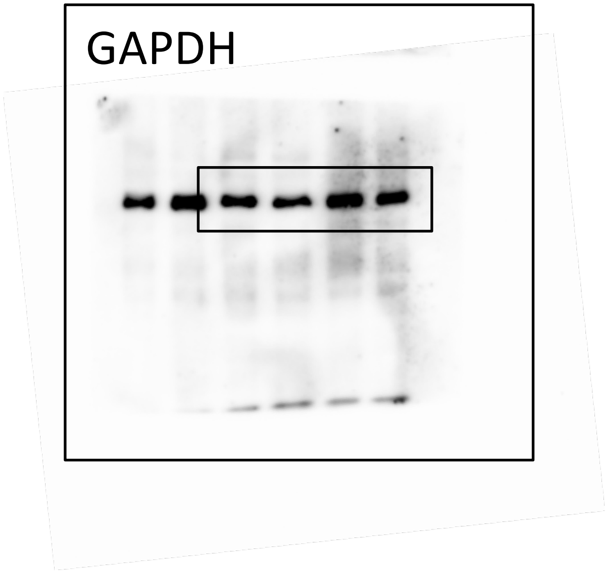

fully unedited gel for Figure 5C
STAT3
JAK1

corresponding Ponceau
stained membranes

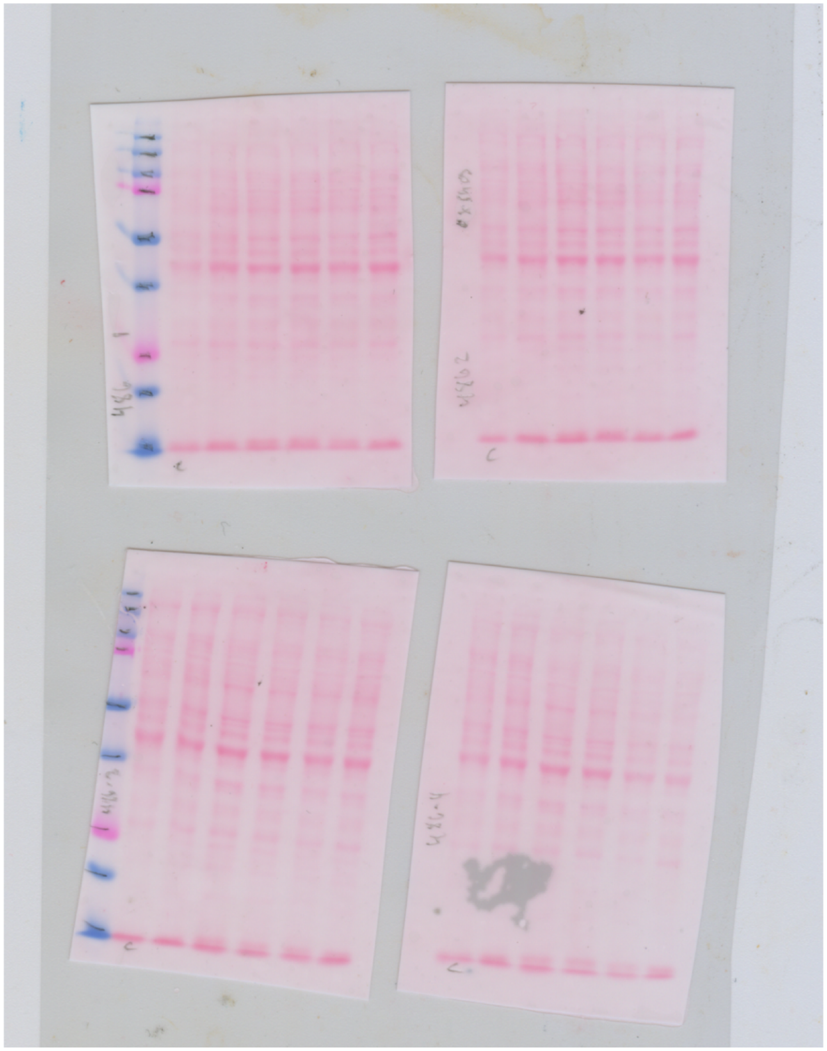

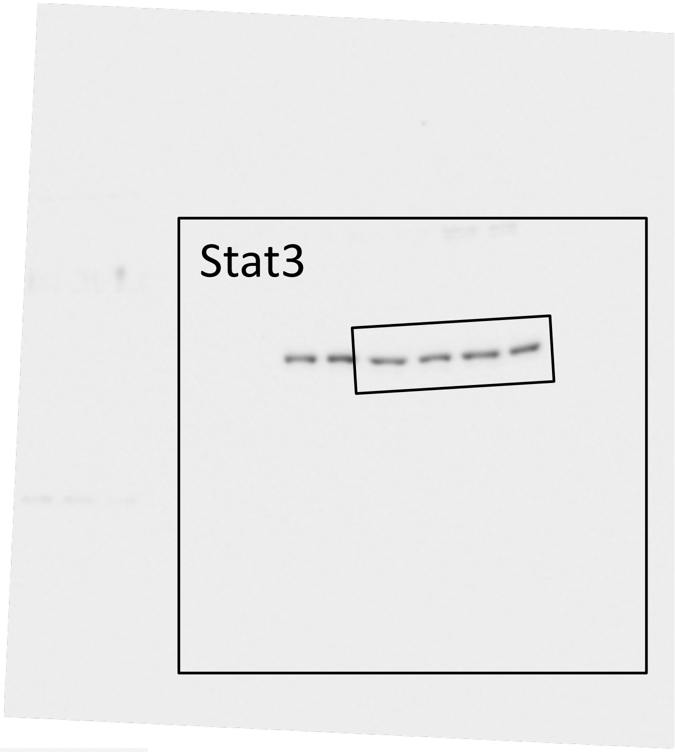

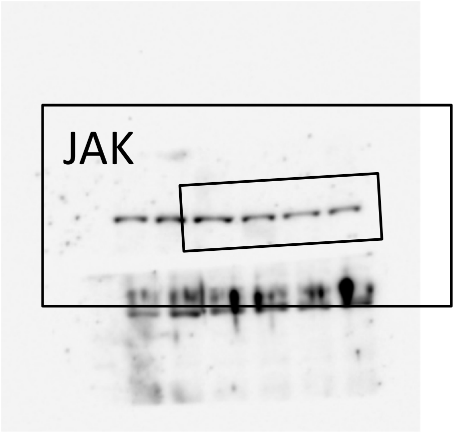

fully unedited membranes for Figure 5D and 6A
NMC transfected with blockIT control (3x) or
mir486 (3x)

corresponding Ponceau
stained membranes

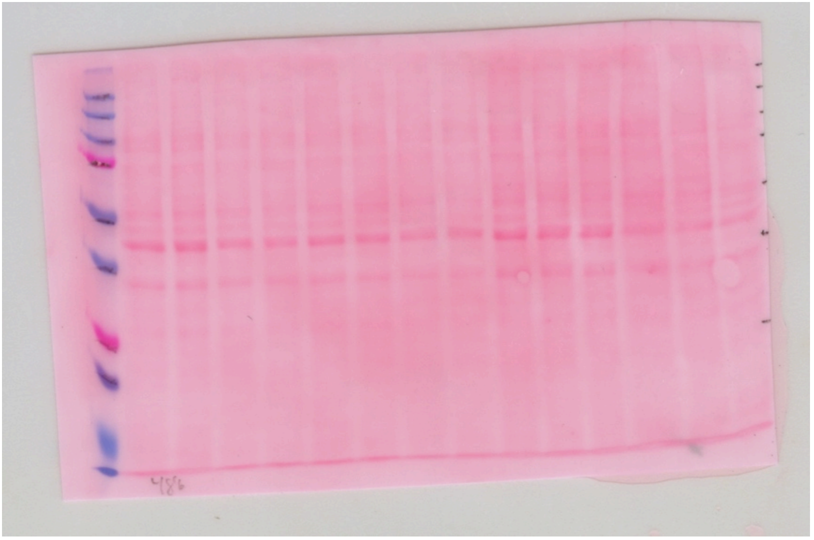

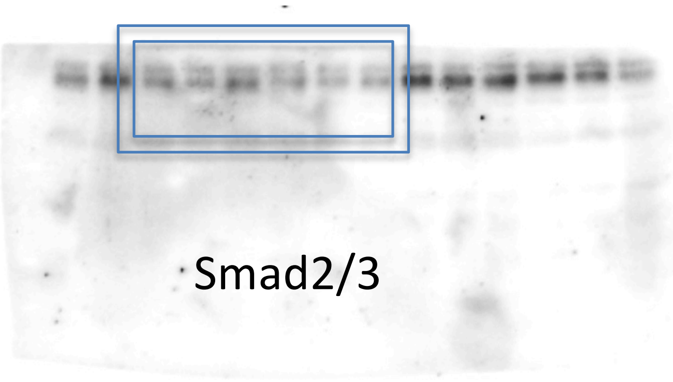

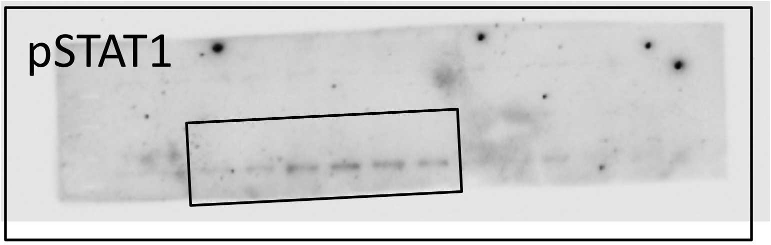

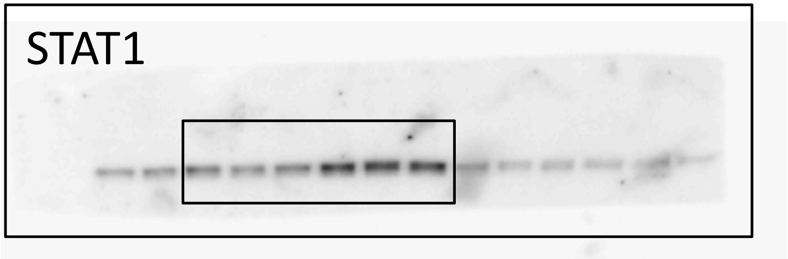

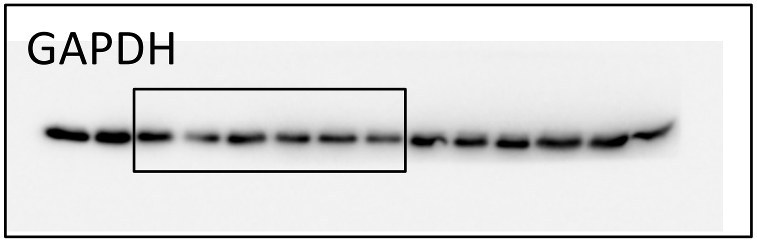

fully unedited gel for Figure 6D
STAT1
pSTAT1 (Ser727)
NMC control (4x) and treated with TGFb2 (3x)

corresponding Ponceau
stained membranes

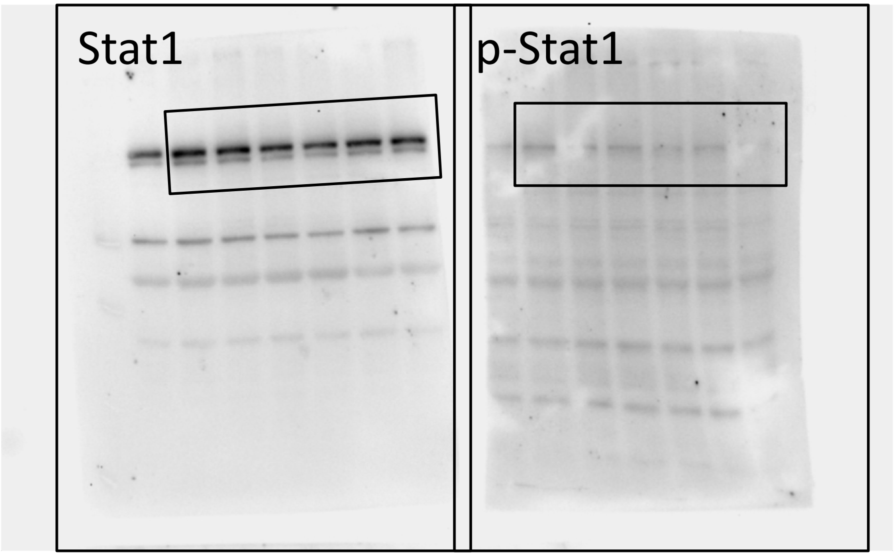

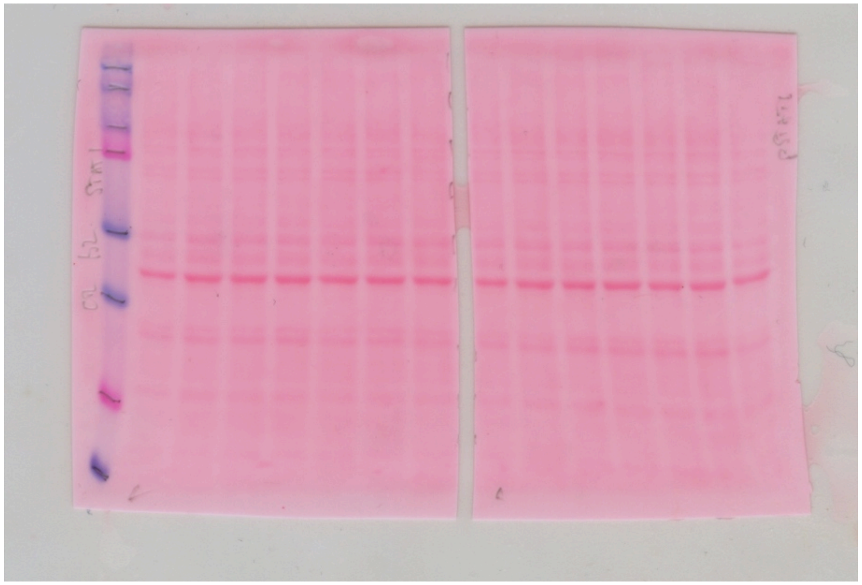

fully unedited gel for Figure 7A
SRF
Gata4
NMC control (3x) and treated with miR486 (3x)

corresponding Ponceau
stained membranes

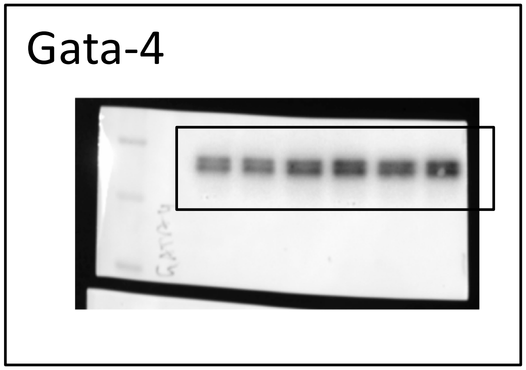

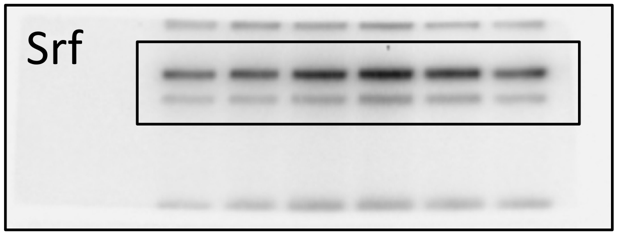

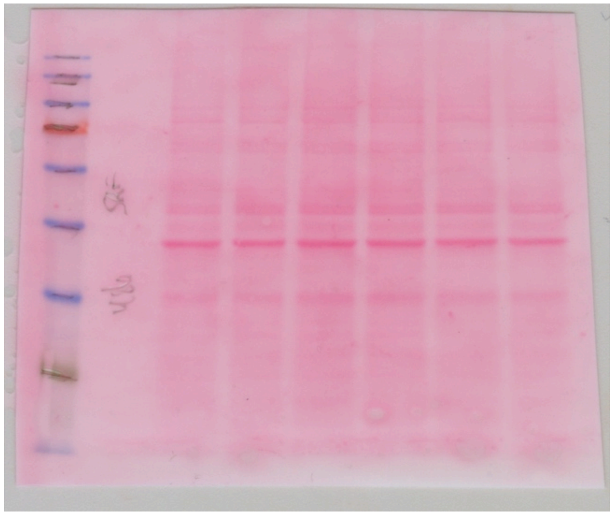

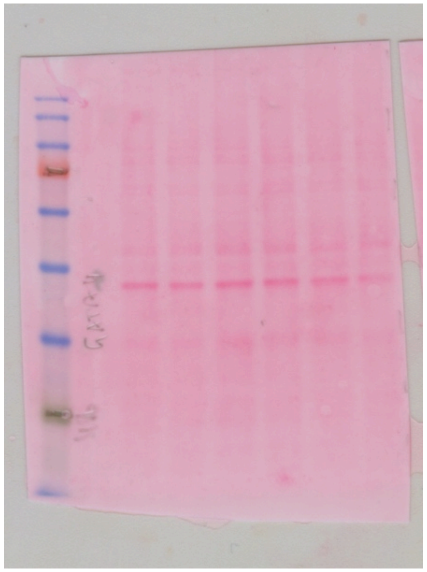

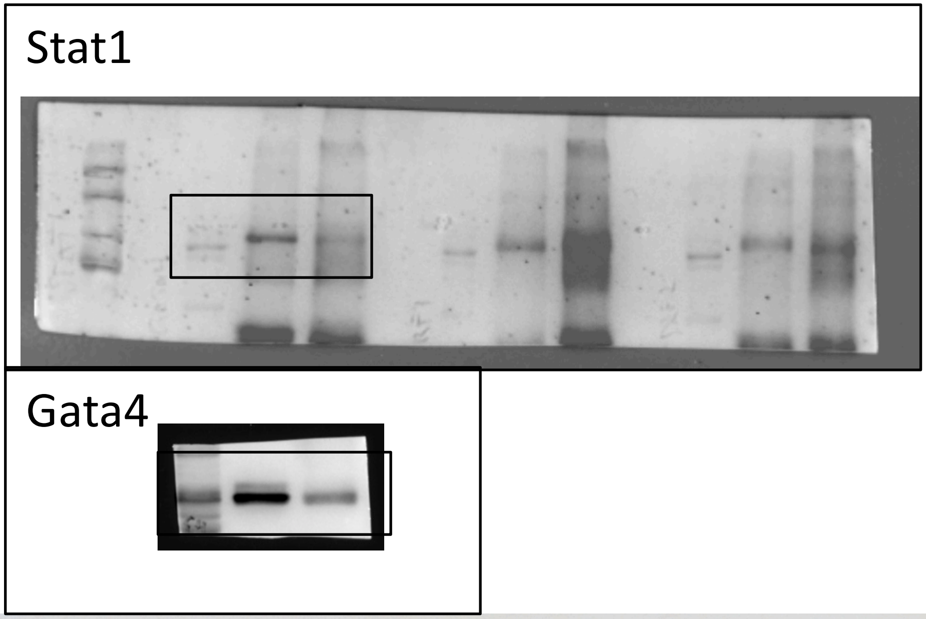

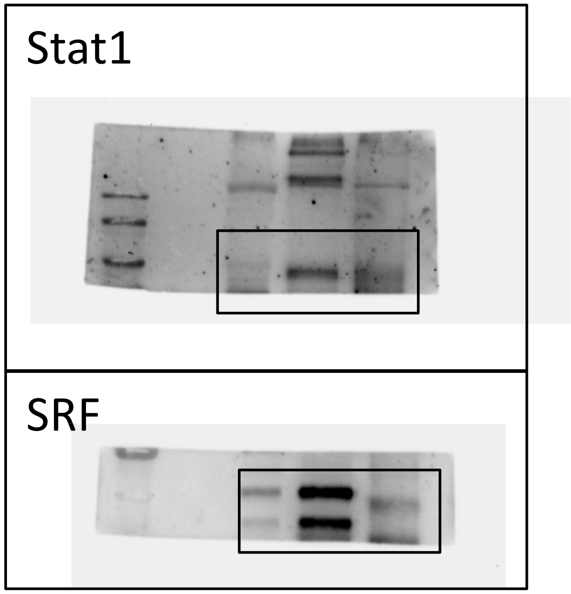

fully unedited gel for
Figure 7B, C
STAT1
IP Gata-4 or SRF
corresponding
Ponceau
stained membranes

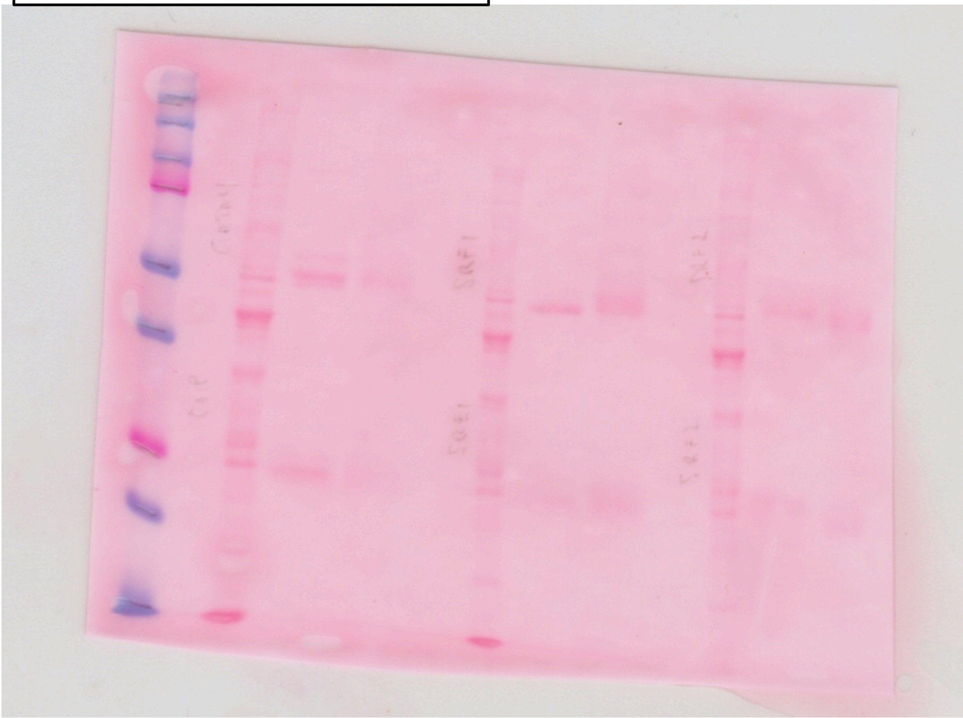

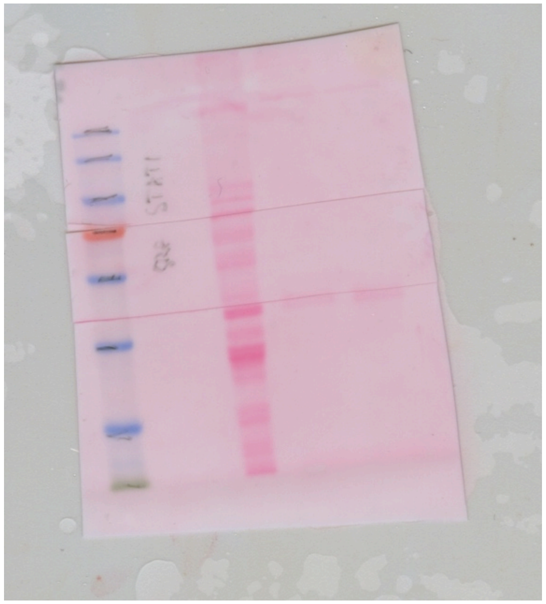

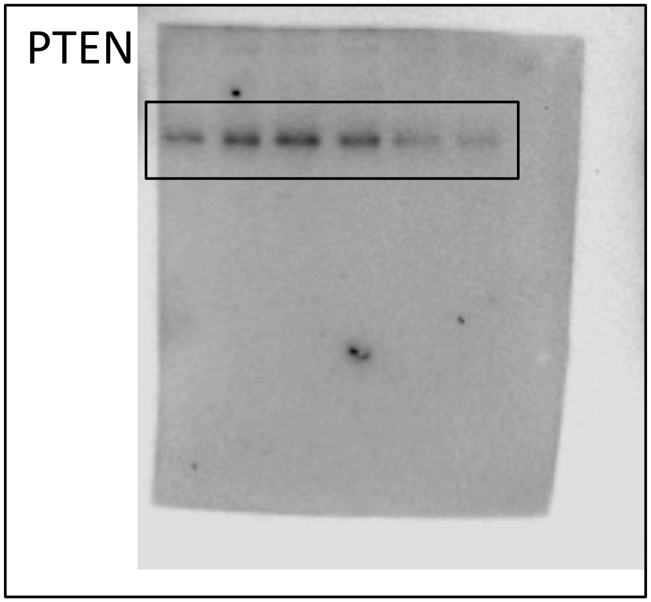

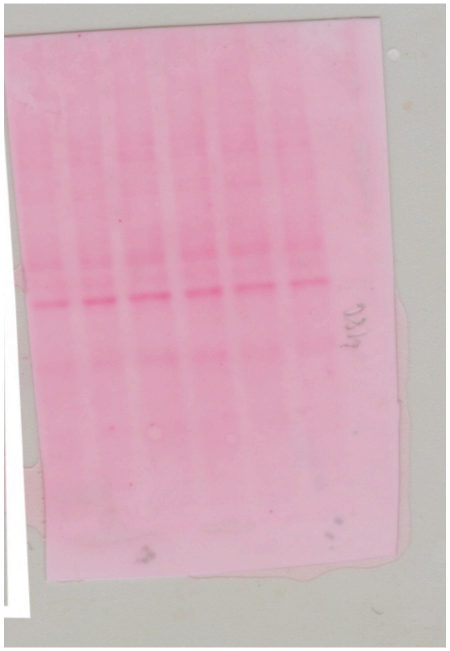

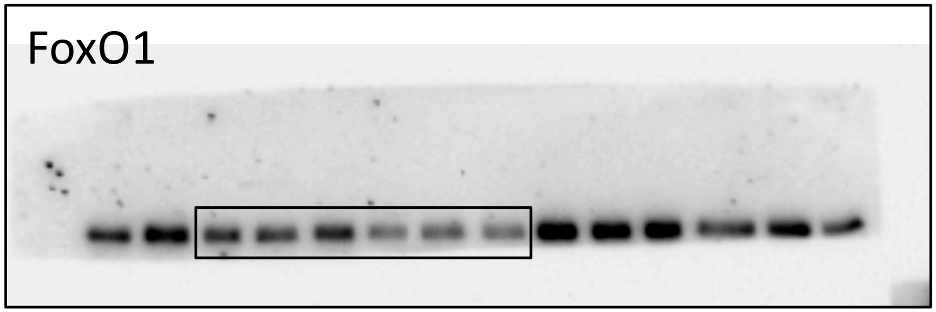

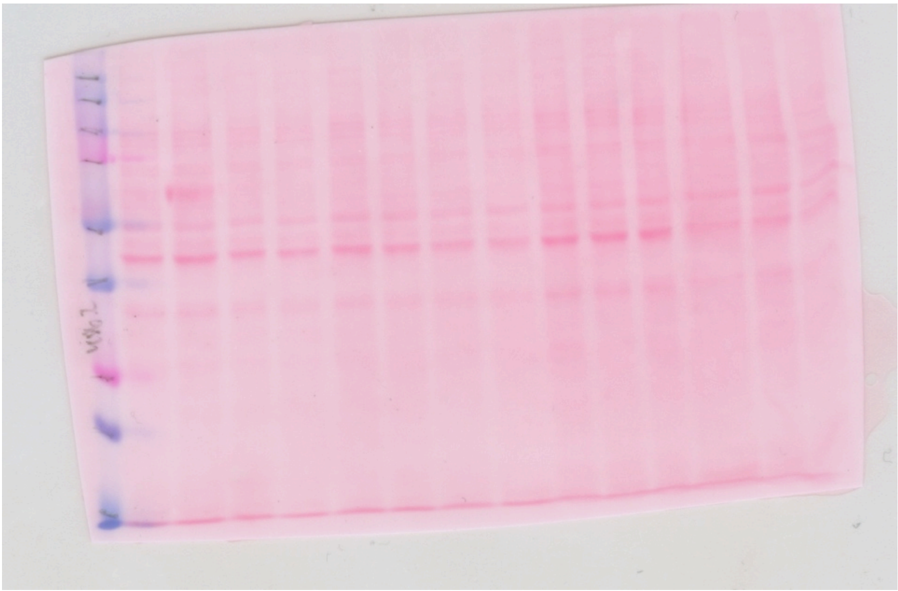

fully unedited gel for Supplemental Figure 1A
PTEN and Foxo-1

NMC control (3x) and treated with miR486 (3x)
corresponding Ponceau stained membranes

titin T11 – shorter exposure

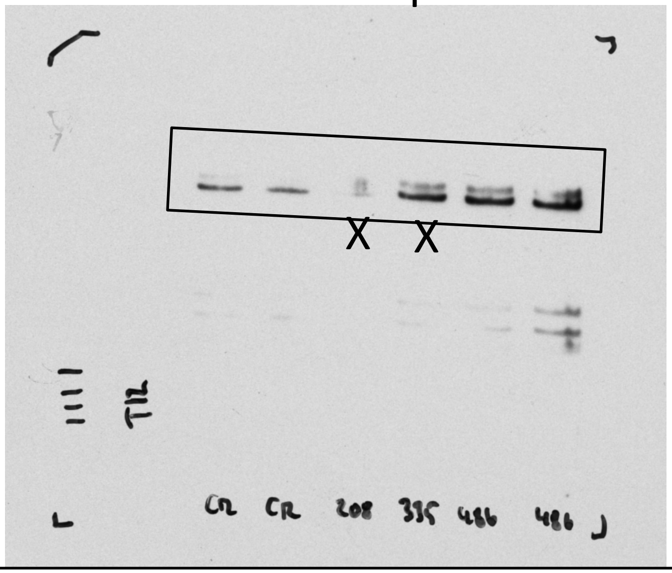

titin T11 – longer exposure

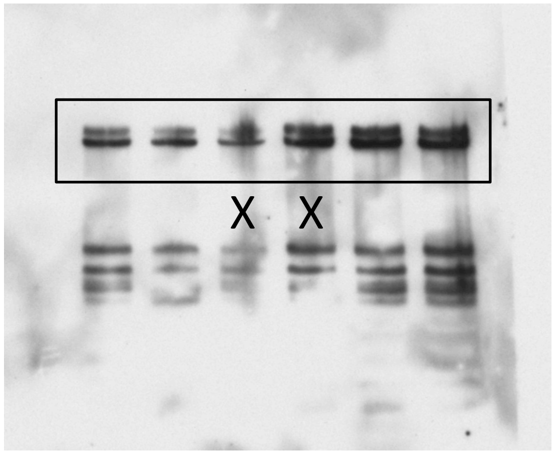

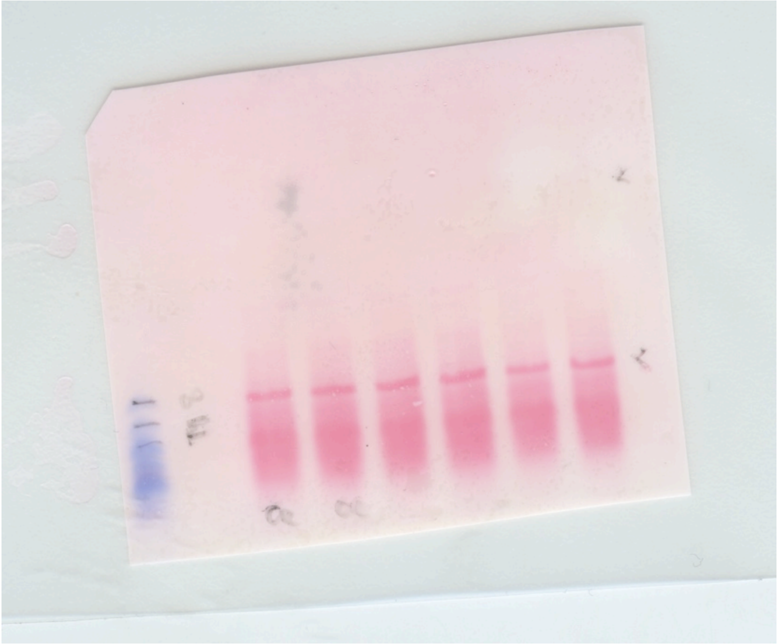

fully unedited gel for Supplemental Figure 1C
titin
NMC control (2x left side) and treated with miR486 (2x right side)
2 center lanes marked with ‘X’ were cut (other miRNA’s)

corresponding Ponceau stained membranes

fully unedited gel for Supplemental Figure 1C
troponin-I, myosin heavy chain, alpha-actinin-2
cardiac actin

NMC control (3x) and treated with miR486 (3x)
corresponding Ponceau stained membranes

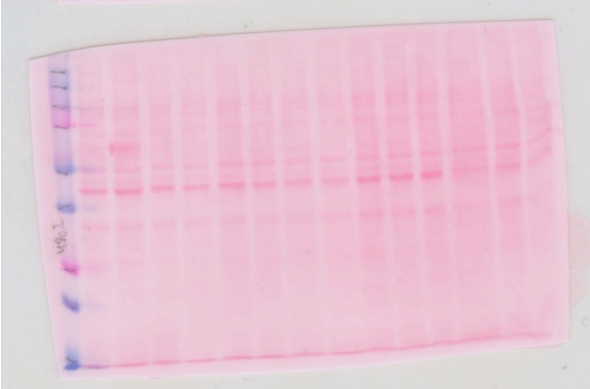

cardiac actin

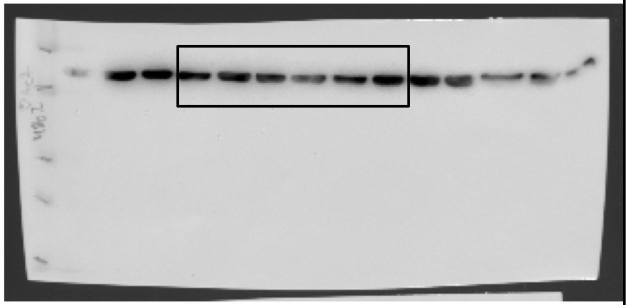

TNI

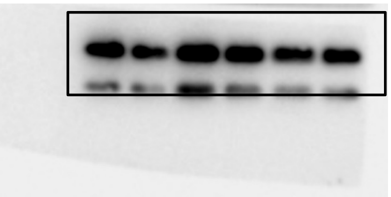

myosin (1025)

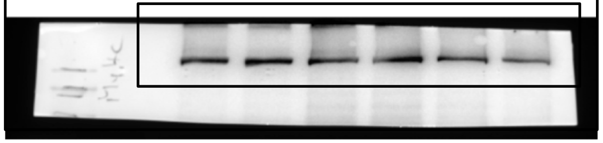

Actinin (sarcomeric)

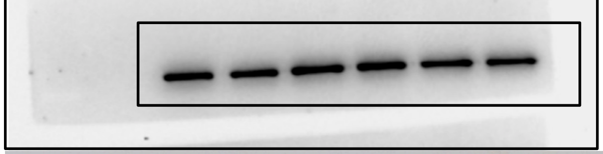

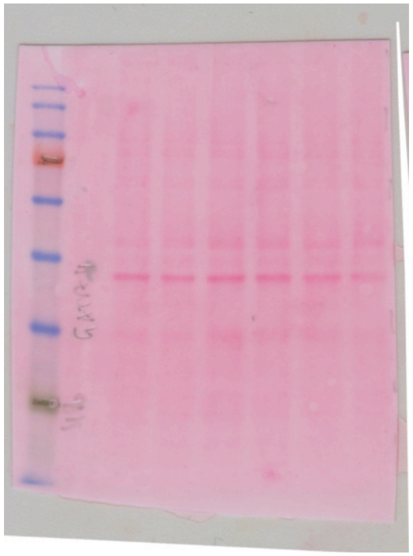

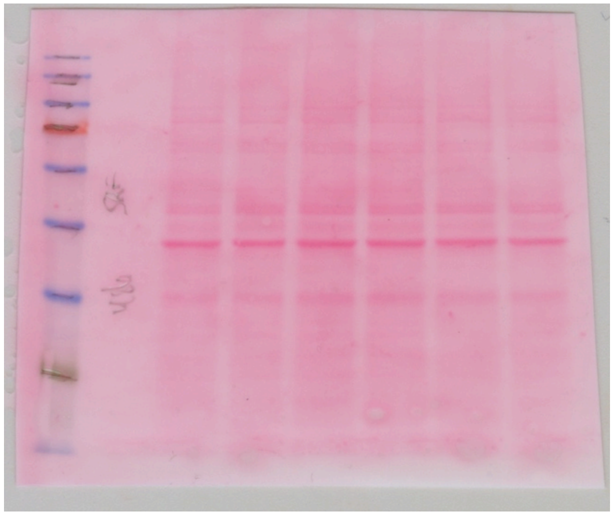

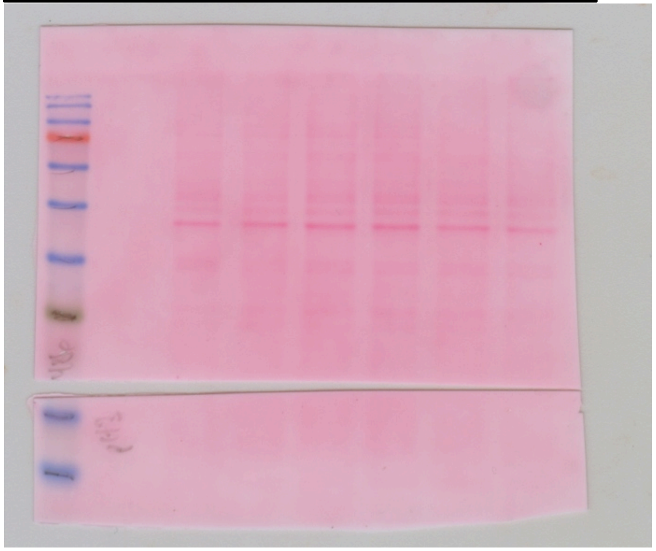

Supplement: Supplemental data [file jciinsight-4-125507-s039.pdf]
